# Anatomical, subset, and HIV-dependent expression of viral sensors and restriction factors

**DOI:** 10.1016/j.celrep.2024.115202

**Published:** 2025-01-10

**Authors:** Ashley F. George, Jason Neidleman, Xiaoyu Luo, Julie Frouard, Natalie Elphick, Kailin Yin, Kyrlia C. Young, Tongcui Ma, Alicer K. Andrew, Ifeanyi J. Ezeonwumelu, Jesper G. Pedersen, Antoine Chaillon, Magali Porrachia, Brendon Woodworth, Martin R. Jakobsen, Reuben Thomas, Davey M. Smith, Sara Gianella, Nadia R. Roan

**Affiliations:** 1 Gladstone Institutes, San Francisco, CA, USA; 2 Department of Urology, UCSF, San Francisco, CA, USA; 3 Department of Biomedicine, Aarhus University, Aarhus, Denmark; 4 Division of Infectious Diseases and Global Public Health, UCSD, La Jolla, CA, USA; 5 Lead contact

## Abstract

We developed viral sensor and restriction factor-cytometry by time of flight (VISOR-CyTOF), which profiles 19 viral sensors and restriction factors (VISORs) simultaneously in single cells, and applied it to 41 postmortem tissues from people with HIV. Mucosal myeloid cells are well equipped with SAMHD1 and sensors of viral capsid and DNA while CD4^+^ T cells are not. In lymph node CD4^+^ Tfh, VISOR expression patterns reflect those favoring integration but blocking HIV gene expression, thus favoring viral latency. We also identify small subsets of bone marrow-, lung-, and gut-associated CD4^+^ T and myeloid cells expressing high levels of restriction factors targeting most stages of the HIV replication cycle. *In vitro*, HIV preferentially fuses to CD4^+^ T cells with a permissive VISOR profile, but early induction of select VISORs by T1IFN prevents productive HIV infection. Our findings document the diverse patterns of VISOR profiles across tissues and cellular subsets and define their association with susceptibility to HIV.

## In brief

George et al. profile viral sensor and restriction factor (VISOR) expression in tissues of people with HIV. Tissue- and subset-dependent VISOR expression was observed, with the highest expression within the mucosa. *In vitro*, HIV enters CD4^+^ T cells but not myeloid cells exhibiting a permissive state, and pre-infection upregulation of VISORs is required for restriction.

## Graphical abstract

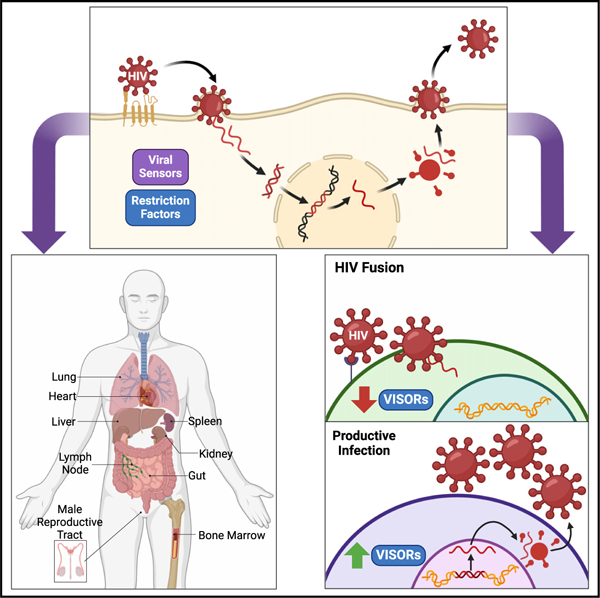

## INTRODUCTION

The fate of a cell exposed to human immunodeficiency virus (HIV) depends on its ability to detect the presence of virus and limit its replication. Host cells are equipped with a collection of pattern recognition receptors (PRRs)^1^ that can recognize viral components such as nucleic acids, proteins, and glycans (collectively referred to as pathogen-associated molecular patterns, or PAMPs). Upon viral sensing, the host cell activates intracellular signaling cascades, leading to the production of interferons (IFNs) and other inflammatory cytokines, which play important roles in controlling viral replication. In particular, type I IFNs (T1IFNs) upregulate hundreds of IFN-stimulated genes (ISGs), which elicit a variety of antiviral functions. Some of these ISGs are restriction factors, a structurally and functionally diverse class of proteins that can directly inhibit almost every step of the HIV replication cycle.^2,3^ Some restriction factors, such as IFI16 (IFN-g-inducible protein 16),^4,5^ directly interact with viral nucleic acid components, thereby also acting as PRRs to further propagate antiviral immune responses. Therefore, viral sensors and restriction factors are crucial first-line agents against viral pathogens. However, the majority of studies identifying and characterizing these proteins were done in cell lines, and little is known about their expression in primary immune cells.

Primary host cells for HIV include both myeloid and CD4^+^ T cells. Myeloid cells play crucial roles in surveillance and defense against pathogens, and their relative resistance to productive infection by HIV compared to their CD4^+^ T cell counterparts is attributed, in part, to higher levels of the restriction factor SAM domain and HD domain-containing protein 1 (SAMHD1). SAMHD1 limits reverse transcription by bringing the pool of available deoxynucleotide triphosphates below the threshold required for HIV cDNA synthesis.^6,7^ Additionally, SAMHD1 has demonstrated exonuclease activity against single-stranded DNAs (ssDNAs) and single-stranded RNAs (ssRNAs),^8^ and this activity can play an important role in degrading the HIV RNA of incoming viral particles.^9^ Although SAMHD1 has been shown to be expressed in most tissues among cells of hematopoietic origin,^10^ its relative expression levels among myeloid and CD4^+^ T cells in different tissue compartments have not been interrogated. Furthermore, the expression patterns of other restriction factors or sensors of HIV have not been interrogated in a cross-tissue manner. One might imagine that mucosal sites with resident microbiomes express a different collection of viral sensors due to constant exposure to environmental or commensal pathogens. Viral sensor expression may also be elevated at tissue sites harboring a high burden of HIV-infected cells (e.g., the gut) due to constant exposure to viral gene products. Although immuno-phenotyping studies have been performed using postmortem human tissues,^11–15^ to date no studies have examined expression of viral sensors and restriction factors across tissue sites.

CyTOF (cytometry by time of flight) offers a useful platform to perform such comprehensive comparative studies because of its ability to simultaneously quantitate a wide variety of proteins at the single-cell level. Unlike single-cell RNA sequencing (which analyzes mRNA gene expression) or cellular indexing of transcriptomes and epitopes (CITE-seq, which analyzes cell-surface protein expression through DNA-barcoded antibodies),^16^ CyTOF allows for in-depth quantitation of proteins that are intracellular, which most viral sensors and restriction factors are. The CyTOF technology has been used not only for deep phenotyping of immune subsets but also for assessing protein phosphorylation states^17^ and glycan features^18^ of immune cells. Here, we established a new CyTOF panel named viral sensor and restriction factor-CyTOF (VISOR-CyTOF), which, in addition to allowing basic phenotyping of immune cells, includes metal-conjugated antibodies against 19 intracellular viral sensing proteins of relevance for HIV. We applied VISOR-CyTOF to 41 tissue specimens obtained postmortem from 7 people with HIV (PWH) and to *in vitro* HIV-infected CD4^+^ T cells and myeloid cells. Our findings suggest differential expression of sensors and restriction factors in a tissue site-, cell type-, and infection-dependent manner and identify multiple instances of insufficient restriction of HIV replication even in cells expressing high levels of HIV restriction factors.

## RESULTS

### Development and validation of VISOR-CyTOF

We developed VISOR-CyTOF to simultaneously phenotype and quantitate the levels of 19 different viral sensors and restriction factors of relevance for HIV infection among immune cells (Figure S1; Table S1). We selected both direct and indirect sensors of viral gene products. These consisted of sensors of HIV capsid (PQBP1 and MX2), RNA (RIGI), and DNA (TLR9, AIM2, IFI16, and cGAS), and downstream effectors of this sensing (pSTING, pIRF3, MX1, and IFIT3); all of these factors have been reported to sense or respond to HIV infection.^1,19–24^ The restriction factors we selected act throughout the HIV replication cycle, including during reverse transcription (SAMHD1 and PAF1), nuclear import (MX2), integration (TRIM28), HIV transcription (TRIM28, BRD4, and IFI16), and HIV translation (IFIT1, IFITM1, and SLFN11); all of these factors have been reported to have the ability to restrict HIV.^2,20,21,23,25,26^ In addition to this collection of viral sensors and restriction factors, which we hereafter refer to as VISORs, our CyTOF panel includes basic phenotyping markers to identify B cells (CD19), myeloid cells (CD14), and T cells (CD3, CD8, and CD4) (Table S2). As CD4^+^ T cells are the primary cellular targets for HIV, our panel also includes the ability to differentiate the major CD4^+^ T cell subsets, including CD4^+^ naive T cells (Tn, CD45RA^+^CD45RO^–^) and memory T cells (Tm, CD45RO^+^ CD45RA^–^), and, within Tm, T central memory cells (Tcm, CD27^+^CCR7^+^), T transitional memory cells (Ttm, CD27^+^CCR7^–^), T effector memory cells (Tem, CD27^–^CCR7^–^), T follicular helper cells (Tfh, PD1^+^CXCR5^+^), regulatory T cells (Treg, CD25^+^ CD127^–^), and CD69^+^ Tm as a proxy for T resident memory (Trm) cells.^12,13,27^ Because HIV preferentially replicates in activated CD4^+^ T cells^28–30^ and persists in CD4^+^ T cells expressing immune checkpoint molecules,^31–33^ we also included markers to identify activation (CD38, CD25, CD69, and HLADR) and checkpoint/exhaustion (PD1, LAG3, and TIGIT) states. Lastly, because most of the HIV reservoir persists in tissues,^34^ we included several tissue-homing markers (CCR5, CCR7, and CXCR5) (Table S2).

We validated the immunophenotyping antibodies by demonstrating the expected differential expression patterns between B and T cells of tonsillar origin (Figure S2A).^18,35^ For validation of VISOR staining, we leveraged prior observations that myeloid-lineage cells (including monocytes) express high levels of viral sensors and restriction factors such as SAMHD1^36^ and STING,^37,38^ relative to B cells. Consistent with these reports, VISOR-CyTOF analysis of peripheral blood mononuclear cells (PBMCs) revealed high SAMHD1 and activated STING (pSTING) expression in monocytes relative to B cells (Figure S2B). Likewise, nearly all other restriction factors and viral sensors included in our panel were elevated in monocytes compared to B cells (Figure S2B). The exceptions were AIM2, IFIT3, and MX1, whose levels did not differ (Figure S2C). But since HIV infection of myeloid cells upregulates AIM2, IFIT3, and MX1,^39,40^ we infected monocytes with HIV and confirmed that VISOR-CyTOF could detect the expected increased expression of these factors (Figure S2C). Together, these data validate VISOR-CyTOF as a tool to monitor expression of viral sensors and restriction factors in primary cells.

### VISOR expression profiles differ between immune subsets

**Figure 1.**
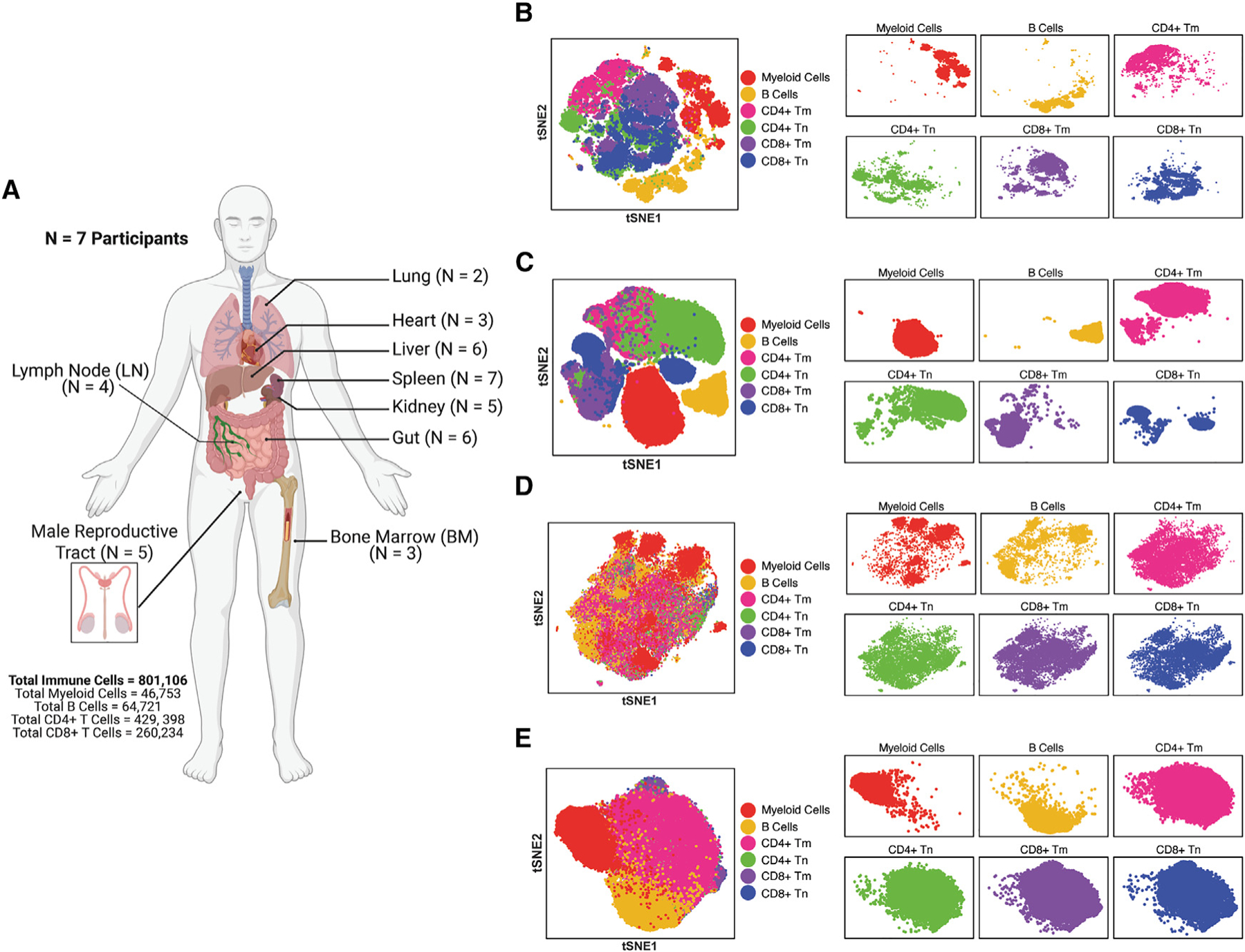
Immune subsets from tissues and blood exhibit distinct VISOR expression patterns (A) Spleen, lymph node (LN), gut, male reproductive tract, kidney, liver, bone marrow (BM), heart, and lung were harvested postmortem from antiretroviral therapy (ART)-treated people with HIV (PWH; *N* = 7) and analyzed by VISOR-CyTOF. Sample sizes for individual tissues are indicated. Total numbers of immune, myeloid, B cells, CD4^+^ T, and CD8^+^ T cells analyzed are shown. Created with BioRender.com. (B and C) VISOR-CyTOF distinguishes myeloid, B, and naive and memory CD4^+^ and CD8^+^ T cells from tissues and blood. Immune cells from the tissues of PWH as described in panel (A) (B) and PBMCs from people without HIV (PWOH; *N* = 4 participants) (C) were phenotyped by VISOR-CyTOF and visualized by tSNE using all markers in the panel. (D and E) tSNE analysis using only VISOR markers of VISOR-CyTOF separates myeloid, B, and T cells from one another. Data from the tissues from PWH (D) and PBMCs from PWOH (E) are shown. In (B)–(E), overlaid tSNEs are shown on the left, while tSNEs separated out by each immune subset are shown on the right. Abbreviations: Tm, memory T cells; Tn, naive T cells.

To establish a holistic view of VISOR expression among immune cells throughout the human body, we obtained fresh, postmortem tissues collected through rapid research autopsy from PWH (*N* = 7) enrolled as part of the Last Gift Program, which comprises individuals approaching the end of life who donate their bodies for HIV cure research. A total of 41 tissue specimens from 9 different tissue sites (spleen, lymph node [LN], gastrointestinal tract [gut], male reproductive tract, liver, kidney, bone marrow [BM], heart, and lung), were processed into single-cell suspensions (STAR Methods) and analyzed by VISOR-CyTOF (Figure 1A). In total, 801,106 live, singlet immune cells were analyzed, including 46,753 myeloid cells, 64,721 B cells, 429,398 CD4^+^ T cells, and 260,234 CD8^+^ T cells (Figure 1A). As blood was not readily available from Last Gift participants, we processed PBMCs from people without HIV (PWOH, *N* = 4) to enable VISOR analysis of peripheral immune cells.

Implementing both VISORs and phenotyping markers for tSNE visualization led to segregation of all the subsets from one another in both tissues (Figure 1B) and blood (Figure 1C). This was expected due to subset-specific expression patterns of our phenotyping markers and is consistent with prior reports.^17,41^ Interestingly, tSNE analysis using only VISOR markers also segregated subsets from one another, albeit less distinctly: while myeloid cells, B cells, and T cells segregated on the tSNE, the four T cell subsets (CD4^+^ Tm, CD4^+^ Tn, CD8^+^ Tm, and CD8^+^ Tn) resided in similar regions (Figures 1D and 1E). These results demonstrate that VISOR profiles globally differ between immune subsets and are more similar between different subsets of T cells than between T cells and non-T cells.

### VISORs including SAMHD1 are highly expressed in myeloid cells and weakly expressed in T and B cells in both tissues and blood

**Figure 2.**
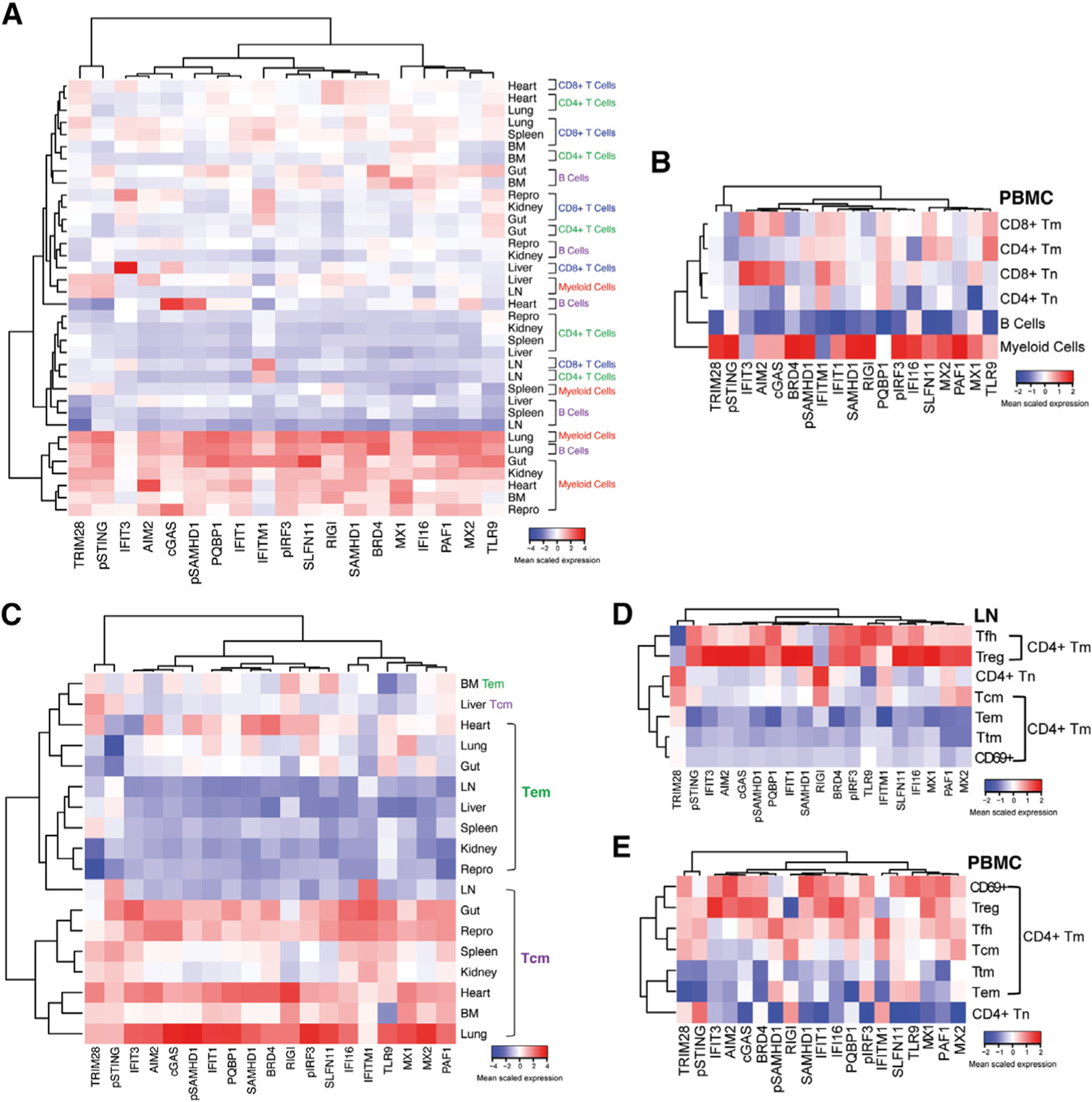
Myeloid cells, Tcm, and Treg express high levels of VISORs while Tem and CD69+ Tm do not (A and B) VISORs are expressed at the highest and lowest levels in myeloid and B cells, respectively, with T cells exhibiting intermediate expression. Results from tissues of PWH (A) and PBMCs of PWOH (B) are shown. (C–E) VISOR expression patterns differ in CD4^+^ T cell subsets. VISORs are more weakly expressed in CD4^+^ Tem relative to Tcm across tissues of PWH (C). In LN of PWH, VISORs are highly expressed in CD4^+^ Tfh, Treg, and Tcm and lowly expressed in Tem, Ttm, and CD69^+^ Tm (D). In PBMCs of PWOH, VISORs are highly expressed in CD4^+^ Treg, Tfh, and Tcm and lowly expressed in Ttm and Tem; low VISOR expression among CD69^+^ Tm likely reflects their being activated cell instead of true Trm (E). For all heatmaps, color intensity (blue to red) denotes the column-normalized mean-scaled expression of each indicated VISOR within the indicated cell subset. Data were generated by unsupervised clustering. Abbreviations: CD4^+^ Tcm, CD4^+^ T central memory; CD4^+^ Tem, CD4^+^ T effector memory; CD4^+^ Ttm, CD4^+^ T transitional memory; CD4^+^ Tfh, CD4^+^ T follicular helper; CD4^+^ Treg, CD4^+^ T regulatory; CD4^+^ CD69^+^ Tm, CD69^+^ T memory as a proxy for T resident memory (Trm) cells.

To assess the drivers behind these differential VISOR expression patterns, and to directly compare expression of individual VISORs between immune cell subsets, we conducted unsupervised hierarchical clustering. Myeloid cells overall expressed higher VISORs than did B cells, CD4^+^ T cells, and CD8^+^ T cells across tissues, and those from the lung, gut, kidney, heart, BM, and male reproductive tract expressed higher levels than did those from the liver, spleen, and LNs (Figure 2A). Notably, SAMHD1, a major HIV restriction factor in myeloid cells,^6,7^ was highly expressed among myeloid cells from most tissue sites. The exceptions were spleen and LNs, suggesting that myeloid cells at these sites may be more permissive to HIV infection. Similar to tissues, VISORs in blood were generally expressed at the highest levels in myeloid cells, intermediary levels in T cells, and the lowest levels in B cells (Figure 2B). Together, our findings demonstrate that, with minor variations, myeloid cells express higher levels of sensors and restriction factors than do T and B cells, in both tissues and blood.

### Differential VISOR profiles among CD4^+^ T cell subsets with implications for HIV latency

CD4^+^ Tm are the main targets of HIV infection, but Tm subsets differ in their relative susceptibilities to HIV. For example, although both Tem and Tcm have been implicated in HIV persistence,^42–45^ Tem are more susceptible to *in vitro* HIV infection than their Tcm counterparts.^41,46^ Unsupervised hierarchical clustering of manually gated CD4^+^ Tcm and Tem across tissue sites revealed that, overall, Tem expressed lower levels of VISORs than did Tcm (Figure 2C), in line with Tem’s increased susceptibility to HIV infection. Other CD4^+^ Tm cell subsets—in particular Trm and Tfh—are also known to be preferentially susceptible to HIV infection.^41,47,48^ We therefore next compared the expression of the classic CD4^+^ Tm cell subsets Tem, Tcm, Ttm, Tfh, Treg, and CD69^+^ Tm from tissues. LNs were the only tissue harboring all these subsets, and comparison of VISOR expression among them in LNs revealed the lowest expression among CD69^+^ Tm (Figure 2D), in line with the high susceptibility of Trm to HIV infection. By stark contrast, CD69^+^ Tm from blood expressed the highest levels of VISORs (Figure 2E). The reason for the high VISOR expression in CD69^+^ Tm from blood but not tissues could be that our Trm marker CD69^12,13,49,50^ also serves as an activation marker in blood,^51^ and hence higher VISOR levels in CD69^+^ Tm from blood could be due to their being activated cells rather than true Trm. Interestingly, CD69^+^ Tm from tissues but not blood are highly susceptible to HIV,^41,48^ which would align with our observed VISOR profiles. In contrast to the CD69^+^ Tm, Treg highly expressed VISORs in both tissues and blood (Figures 2D and 2E), suggesting them to be restrictive for HIV replication in a site-independent manner.

**Figure 3.**
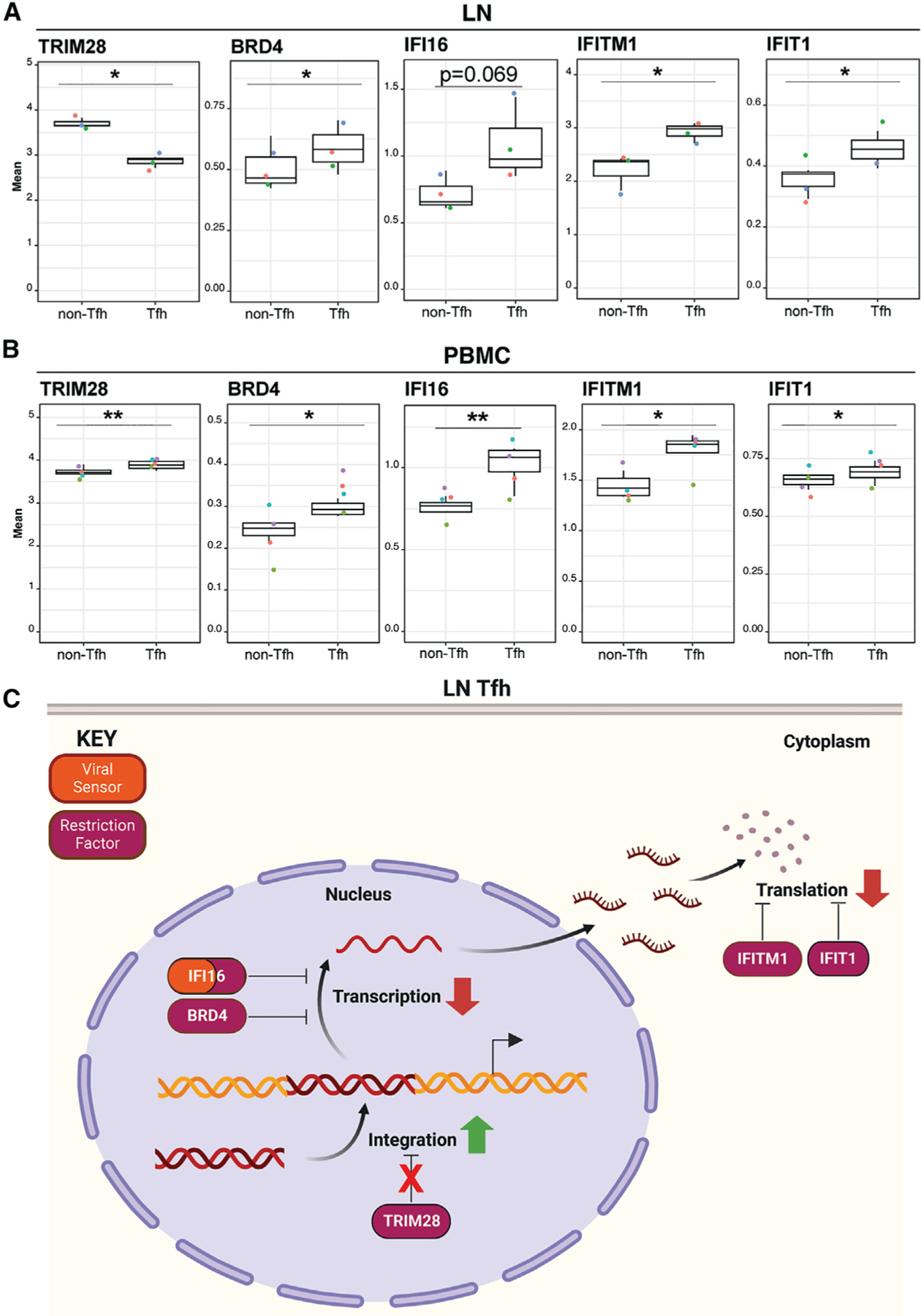
Lymph node CD4^+^ Tfh lowly express integration inhibitor TRIM28 and highly express inhibitors of HIV transcription and translation, potentially favoring HIV latency (A) In LN, restriction factor TRIM28, which blocks HIV integration, is lowly expressed in HIV-permissive CD4^+^ Tfh as compared to non-Tfh, while HIV transcription inhibitors (BRD4 and IFI16) and HIV translation inhibitors (IFITM1 and IFIT1) exhibit the opposite expression pattern. (B) In PBMCs, TRIM28, BRD4, IFI16, IFITM1, and IFIT1 are all more highly expressed in CD4^+^ Tfh as compared to non-Tfh. *p < 0.05, **p < 0.01, as assessed using Student’s two-sided paired t test. Error bars correspond to SD. (C) Proposed model of restriction factor-mediated HIV latency in LN CD4^+^ Tfh. Low expression of TRIM28 in LN CD4^+^ Tfh facilitates HIV integration, while high levels of BRD4, IFI16, IFITM1, and IFIT1 limit HIV gene expression thereby promoting latency. Created with BioRender.com.

Our data also suggested CD4^+^ Tfh to highly express VISORs (Figures 2D and 2E), but this was counterintuitive given that this subset has been reported to be a major target for HIV infection and persistence, particularly within LNs.^47,52^ To examine this more closely, we next compared VISOR expression between CD4^+^ Tfh and CD4^+^ non-Tfh Tm from LNs. This revealed that, although Tfh expressed elevated levels of multiple VISORs, TRIM28, a restriction factor that blocks HIV DNA integration by deacetylating HIV-1 integrase,^53^ was more lowly expressed in Tfh (Figure 3A), which would favor integration among these cells.

As TRIM28 has also been shown to repress HIV transcription in T cells (by facilitating CDK9 SUMOylation resulting in P-TEFb repression^54^), low TRIM28 may also facilitate active gene expression of the integrated HIV provirus. However, we also found that LN CD4^+^ Tfh expressed high levels of BRD4 and IFI16 (Figure 3A), inhibitors of HIV transcription.^55–58^ This observation, together with our observation that restriction factors IFIT1 and IFITM1—both of which inhibit HIV translation^59,60^—were also highly expressed in LN CD4^+^ Tfh (Figure 3A), suggests an overall block in HIV gene expression in these cells. In contrast to CD4^+^ Tfh from LN, those from PBMCs highly expressed TRIM28, suggesting HIV integration would be limited in these cells (Figure 3B). These data together support a model whereby CD4^+^ Tfh from LN but not blood support HIV integration but block HIV gene expression, which would favor establishment of HIV latency (Figure 3C).

### A subset of checkpoint-expressing CD4^+^ T cells enriched in BM and lung expresses high levels of sensors of HIV capsid and DNA and restriction factors targeting most stages of the HIV replication cycle

Having found differences between classic CD4^+^ T cell subsets, we next asked whether VISOR expression correlated with tissue origin or other phenotypic features. We first visualized VISOR expression among total CD4^+^ T cells in all tissues. Overall, highest expression was observed in the heart and lung, followed by BM and gut (Figure S3A). By contrast, CD4^+^ T cells from the spleen, liver, LN, male reproductive tract, and kidney had relatively low VISOR expression (Figure S3A). Next, we performed clustering analysis, which identified four clusters (C1–C4) of CD4^+^ T cells (Figure S3B). Cluster C1 was predominantly Tcm and Ttm, cluster C2 was predominantly Tem, and clusters C3 and C4 were a mix of Tcm Tem, Ttm, and other memory T cells. In addition, cluster C3 was enriched for Treg, and cluster C2 for CD69^+^ Tm (Figure S3C). In terms of anatomical distribution, cluster C1 cells were enriched in the heart, BM, LN, and spleen while cluster C2 cells exhibited the opposite pattern, being more enriched in the gut, male reproductive tract, liver, and kidney. Cluster C3 cells were enriched primarily in the BM and lung. While no significant associations with tissue compartment were found for cluster C4, gut and lung tissues harbored relatively higher frequencies of cluster C4 cells compared to the other tissues (Figure S3D). Among the four clusters, cluster C3 exhibited highest overall VISOR expression; elevated sensors in this cluster included those that sense HIV capsid (MX2) and DNA (AIM2, IFI16, cGAS, and pIRF3), and elevated restriction factors included those targeting reverse transcription (SAMHD1 and PAF1), nuclear import (MX2), transcription (IFI16), and translation (IFITM1, IFIT1, and SLFN11) (Figures S3E and S3F). These findings suggest that cluster C3 cells are equipped with a formidable antiviral defense, as evidenced by their high expression of HIV capsid and DNA sensors, and restriction factors targeting all stages of the HIV replication cycle except integration. Interestingly, cluster C3 cells also expressed high levels of checkpoint molecules PD1, LAG3, and TIGIT (Figure S3G), suggesting that they may be recently activated or exhausted cells. These results demonstrate that, among the four clusters of tissue CD4^+^ T cells identified by clustering, a cluster enriched in the BM and lung that expresses multiple checkpoint molecules is the most restrictive for HIV replication.

### A subset of CCR7+ myeloid cells enriched in the gut exhibits a VISOR profile similar to cluster C3 of CD4^+^ T cells

**Figure 4.**
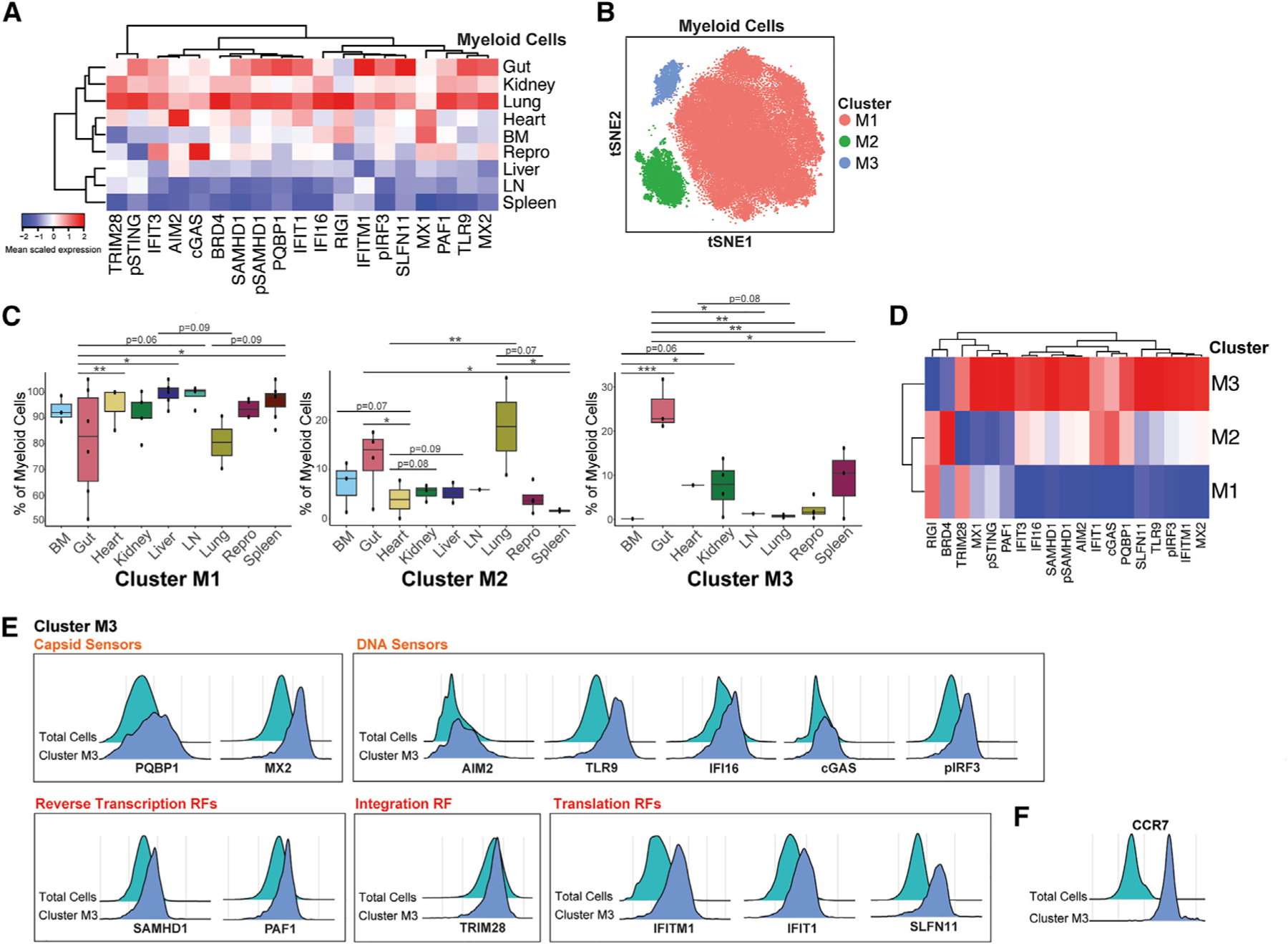
A subset of HIV-restrictive CCR7+ myeloid cells from gut expresses high levels of sensors of HIV capsid and DNA and restriction factors targeting all stages of the HIV replication cycle (A) VISORs are most highly expressed in myeloid cells from the gut, kidney, and lung and most lowly expressed in those from the liver, LN, and spleen. (B) Louvain clustering identifies three clusters of tissue myeloid cells (M1–M3). tSNE plots were generated using all markers in the CyTOF panel. (C) Distribution of myeloid cell clusters across tissue sites. Cluster M1 cells are enriched in BM, heart, kidney, liver, LN, male reproductive tract, and spleen; cluster M2 cells are enriched in the gut and lung; cluster M3 cells are enriched in the gut. Individual points represent the percentage of all myeloid cells from a given participant belonging to the respective cluster. *p < 0.05, **p < 0.01, ***p < 0.001 as assessed using a generalized linear mixed model with multiple correction by Benjamini-Hochberg procedure for false discovery rate. (D) VISORs are most highly expressed in cluster M3 cells. (E) Cluster M3 cells express high levels of sensors recognizing HIV capsid (PQBP1 and MX2) and DNA (AIM2, TLR9, IFI16, cGAS, and pIRF3). Restriction factors, including those targeting HIV reverse transcription (SAMHD1 and PAF1), nuclear import (MX2), integration (TRIM28), transcription (TRIM28 and IFI16), and translation (IFITM1, IFIT1, and SLFN11), are also preferentially expressed in this cluster. (F) Cluster M3 cells express high levels of CCR7, suggesting their identity to be dendritic cells.

We then performed a similar analysis among tissue myeloid cells. VISOR expression was the highest in the gut, kidney, and lung and the lowest in the liver, LN, and spleen (Figure 4A). Unsupervised clustering identified three clusters (clusters M1–M3) of myeloid cells (Figure 4B). Cluster M1 cells were highly abundant in most tissue sites except for the gut and lung, whereas cluster M2 cells were preferentially enriched at these two sites. Cluster M3 cells were found at increased frequencies in the gut (Figure 4C). Among the three clusters, cluster M3 cells exhibited the highest VISOR expression, with a VISOR profile similar to that found for cluster C3 cells from the CD4^+^ T cell clustering. Elevated VISORs in cluster M3 included all those observed in cluster C3, in addition to capsid sensor PQBP1, DNA sensor TLR9, and the HIV integration/transcription restriction factor TRIM28 (Figures 4D and 4E). Interestingly, cluster M3 cells also expressed high levels of the chemokine receptor CCR7 (Figure 4F), suggesting they may be maturing dendritic cells.^61–64^

### Activated CD14^+^CD8^+^ T cells expressing the transcription restriction factor BRD4 are enriched in the gut and lung

While myeloid cluster M3 cells expressed high levels of many VISORs, one exception was BRD4, which was lowly expressed. By contrast, cluster M2 cells uniquely expressed high levels of this transcription restriction factor (Figure S4A). BRD4 is an epigenetic reader that can suppress HIV gene expression,^55,65^ and hence its high expression in cluster M2 cells may restrict HIV replication. Surprisingly, however, cluster M2 cells also expressed intermediate levels of CD8^+^ T cell markers CD3 and CD8 (Figure S4B), despite being initially classified as myeloid cells due to our defining these cells to be CD3^–^ cells expressing CD14 (STAR Methods). Closer examination of our gating strategy revealed that our ‘‘CD3–" population in fact included a small population of CD3^intermediate^ cells (Figure S4C), which also expressed intermediate levels of CD8 (Figure S4D). Recent work has identified a population of CD8^+^ Trm that acquire CD14 from myeloid cells, with imaging flow cytometry confirming that these cells are not T cell/monocyte doublets.^66^ These cells are enriched in human tissues, including in the liver, skin, spleen, and LNs, and exhibit enhanced activation and immunomodulatory characteristics.^66^ Interestingly, these previously described CD14^+^CD8^+^ T cells closely resemble our cluster M2 cells, in that they express high levels of Trm marker CD69, activation markers, and checkpoint molecules,^66^ as observed by our VISOR-CyTOF analysis (Figure S4E). We also found CD14^+^CD8^+^ T cells to express high levels of CCR5, CCR7, and CXCR5 (Figure S4E), which, together with prior reports of their expressing CXCR3 and CXCR4,^66^ suggest these cells to be tissue homing. As CD8^+^ T cells’ acquisition of the CD14 receptor complex appears to be driven by bacterial wall lipopolysaccharide,^66^ it is conceivable that their preferential enrichment in the gut and lung may result from their being induced by the local microbiome communities of these mucosal sites. Together, these results suggest that a previously described subset of activated CD8^+^ T cells that have acquired CD14 expresses high levels of BRD4 and preferentially resides in microbiome-rich mucosal sites. However, we cannot exclude the possibility that cluster M2 cells include doublets of CD14^+^ monocytes bound to CD8^+^ T cells, given that we did not validate the singlet nature of these cells as previously performed using imaging flow cytometry.^66^

### HIV-fused CD4^+^ T cells globally express low levels of HIV sensors and restriction factors, while HIV-fused myeloid cells highly express SAMHD1

We have thus far presented cross-tissue landscapes of VISOR expression among immune cells, with implications for HIV infection and persistence. As HIV-infected cells are rare *in vivo* even in viremic individuals,^67^ we expect the vast majority of the cells from these participants to be uninfected. To directly study the relationship between VISOR expression and HIV susceptibility, we turned to *in vitro* HIV infection assays.

**Figure 5.**
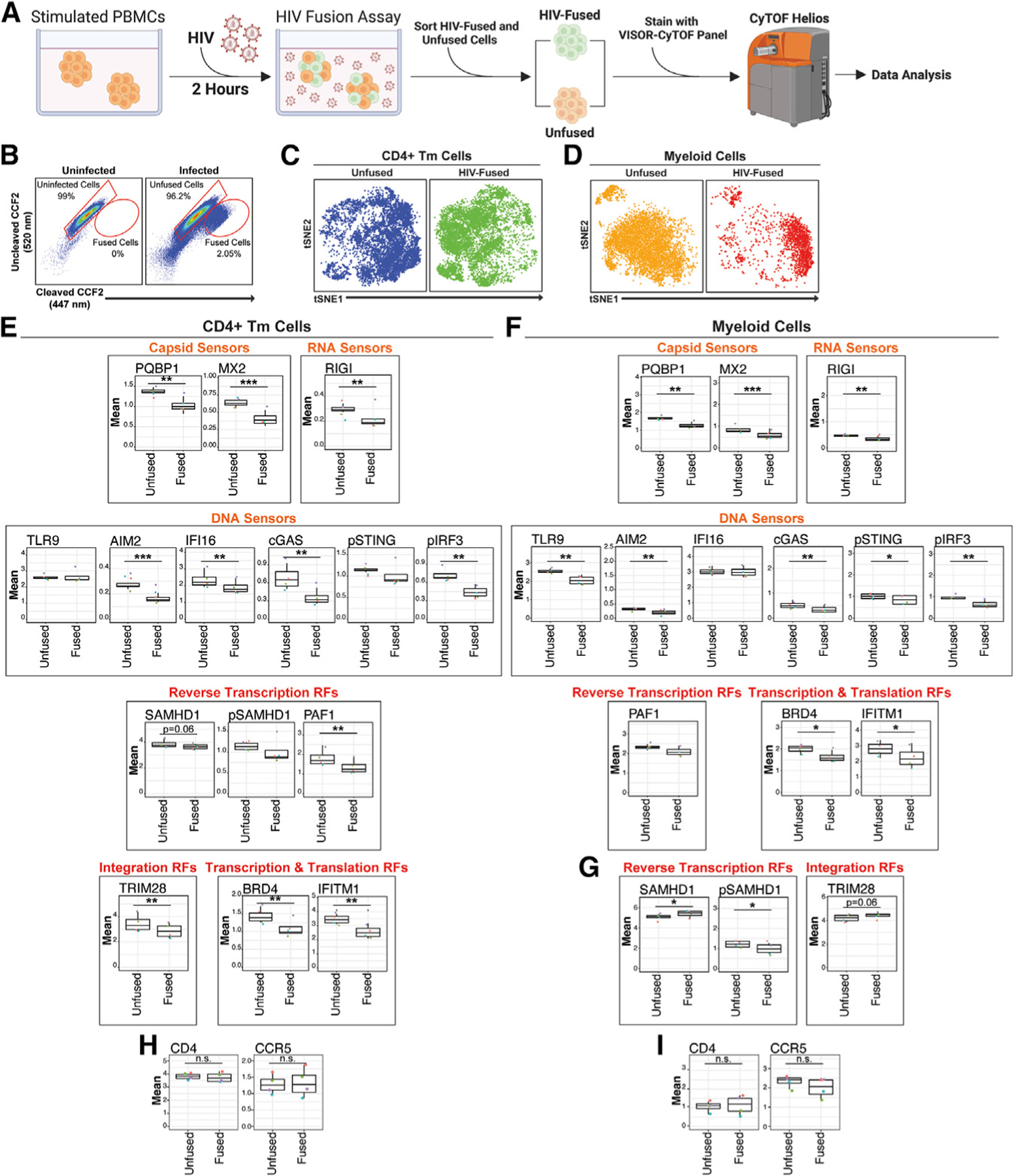
HIV-fused cells express low levels of HIV sensors and restriction factors, with the exception of SAMHD1 and TRIM28 among myeloid cells (A) Fusion assay experimental design. PHA-stimulated, CCF2-loaded PBMCs (*N* = 4) were mock-treated or incubated for 2 h with HIV-F4.HSA virions packaged with Blam-Vpr. Cleavage of CCF2 in HIV-fused cells by Blam-Vpr results in shift from green to blue fluorescence, detectable by FACS. Created with BioRender.com. (B) CCF2 profiles of uninfected and HIV-exposed cultures, which were sorted for unfused and HIV-fused cells and then analyzed by CyTOF-VISOR. Events were pre-gated on live, singlet cells. Gating strategy in Figure S5A. (C and D) tSNE plots of unfused and HIV-fused CD4^+^ Tm (C) or myeloid (D) cells analyzed by VISOR-CyTOF, highlighting dissimilarity of HIV-fused cells to their unfused counterparts. tSNE plots were generated using all markers in the CyTOF panel. (E and F) HIV-fused CD4^+^ Tm and myeloid cells express low levels of multiple VISORs, relative to their unfused counterparts. HIV-fused CD4^+^ Tm (E) and myeloid (F) cells lowly express sensors of HIV capsid (PQBP1 and MX2), RNA (RIGI), and DNA (AIM2, cGAS, and pIRF3). Additionally, HIV DNA sensor IFI16 is lowly expressed in HIV-fused CD4^+^ Tm (E), while HIV DNA sensors TLR9 and pSTING are lowly expressed in HIV-fused myeloid cells (F). With regards to restriction factors, HIV-fused CD4^+^ Tm express low levels of factors targeting HIV reverse transcription (SAMHD1 and PAF1), nuclear import (MX2), integration (TRIM28), transcription (TRIM28, IFI16, and BRD4), and translation (IFITM1) (E). HIV-fused myeloid cells express low levels of restriction factors targeting HIV nuclear import (MX2), transcription (BRD4), and translation (IFITM1) (F). (G) HIV-fused myeloid cells express high levels of SAMHD1 and TRIM28. The phosphorylated and inactive form of SAMHD1 (pSAMHD1) exhibits the opposite expression pattern. (H and I) HIV-fused and unfused cells express equivalent levels of HIV receptor and co-receptor. Mean expression levels of CD4 and CCR5 in unfused and HIV-fused CD4^+^ Tm (H) and myeloid (I) cells. *p < 0.05, **p < 0.01, ***p < 0.001, n.s., non-significant, as assessed using Student’s two-sided paired t tests. Error bars correspond to SD.

We first implemented an HIV fusion assay^68^ to determine the intracellular VISOR landscape of HIV-infected cells during the earliest stage (viral entry) of the HIV replication cycle. Phytohemagglutinin (PHA)-stimulated PBMCs were inoculated with Blam-Vpr-containing HIV-F4.HSA virions to enable identification of HIV-fused cells, and cells supporting fusion (‘‘fused’’) or their unfused counterparts from the virus-exposed culture (‘‘unfused’’) were sorted by fluorescence-activated cell sorting (FACS) (Figure S5A) and analyzed by VISOR-CyTOF (Figures 5A and 5B). Cells from a mock-treated culture never exposed to HIV (‘‘uninfected’’) were sorted and analyzed in parallel. Since HIV preferentially fuses with CD4^+^ Tm rather than Tn,^35^ we restricted our CD4^+^ T cell analysis to Tm cells. By tSNE, fused CD4^+^ Tm (Figure 5C) and myeloid (Figure 5D) cells segregated separately from their unfused counterparts, suggesting that HIV preferentially enters cells with distinct phenotypic features. Strikingly, all VISORs differentially expressed between fused and unfused CD4^+^ Tm were downregulated in the HIV-fused ones (Figure 5E). These downregulated VISORs covered all classes of sensors and restriction factors, in particular sensors of HIV capsid (PQBP1 and MX2), RNA (RIGI), and DNA (AIM2, IFI16, cGAS, and pIRF3) and restriction factors targeting HIV reverse transcription (SAMHD1 and PAF1), nuclear import (MX2), integration (TRIM28), transcription (TRIM28, IFI16, and BRD4), and translation (IFITM1) (Figure 5E). When we compared fused cells to uninfected cells (instead of to unfused cells), we found that the same VISORs were expressed at lower levels among the fused cells (Figure S6A). However, several of these (PAF1, SAMHD1, and TRIM28) no longer had statistical significance, likely because the uninfected cell population is a more heterogeneous population that includes cells that might or might not have allowed for fusion.

Similarly, many VISORs were downregulated in fused myeloid cells relative to their unfused counterparts; these included sensors of HIV capsid (PQBP1 and MX2), RNA (RIGI), and DNA (TLR9, AIM2, cGAS, pSTING, and pIRF3) and restriction factors targeting HIV nuclear import (MX2), transcription (BRD4), and translation (IFITM1) (Figure 5F). Similar results were observed when comparing fused to uninfected myeloid cells, but the differences for cGAS, pSTING, pIRF3, MX2, BRD4, and IFITM1 no longer reached statistical significance (Figure S6B). Notably, however, fused myeloid cells expressed higher levels of restriction factors SAMHD1 and TRIM28, which target HIV reverse transcription and integration, respectively, as compared to both unfused cells and uninfected cells (Figures 5G and S6B). That the phosphorylated (and therefore inactive^9,69^) form of SAMHD1 (pSAMHD1) was downregulated within fused myeloid cells (Figure 5G) further supported the notion of elevated SAMHD1 activity within these cells.

Together, these data demonstrate that, while CD4^+^ Tm and myeloid cells with diminished host defenses are preferential targets for HIV fusion, fused myeloid cells uniquely maintain some restrictive properties—in particular elevated SAMHD1 and TRIM28 expression—which may allow them to remain relatively resistant to productive HIV infection. To interrogate whether the ability of HIV to preferentially fuse to target cells with overall low VISOR expression was due to these cells preferentially expressing high levels of the HIV receptor and co-receptor, we compared expression of CD4 and CCR5 between fused and unfused cells. This revealed that expression levels of both CD4 and CCR5 were equivalent between these populations (Figures 5H and 5I). Therefore, the preferential fusion of HIV with target cells with overall low VISOR expression cannot be accounted for by HIV receptor/co-receptor expression levels.

### Productively infected cells exhibit heightened expression of viral sensors and restriction factors

**Figure 6.**
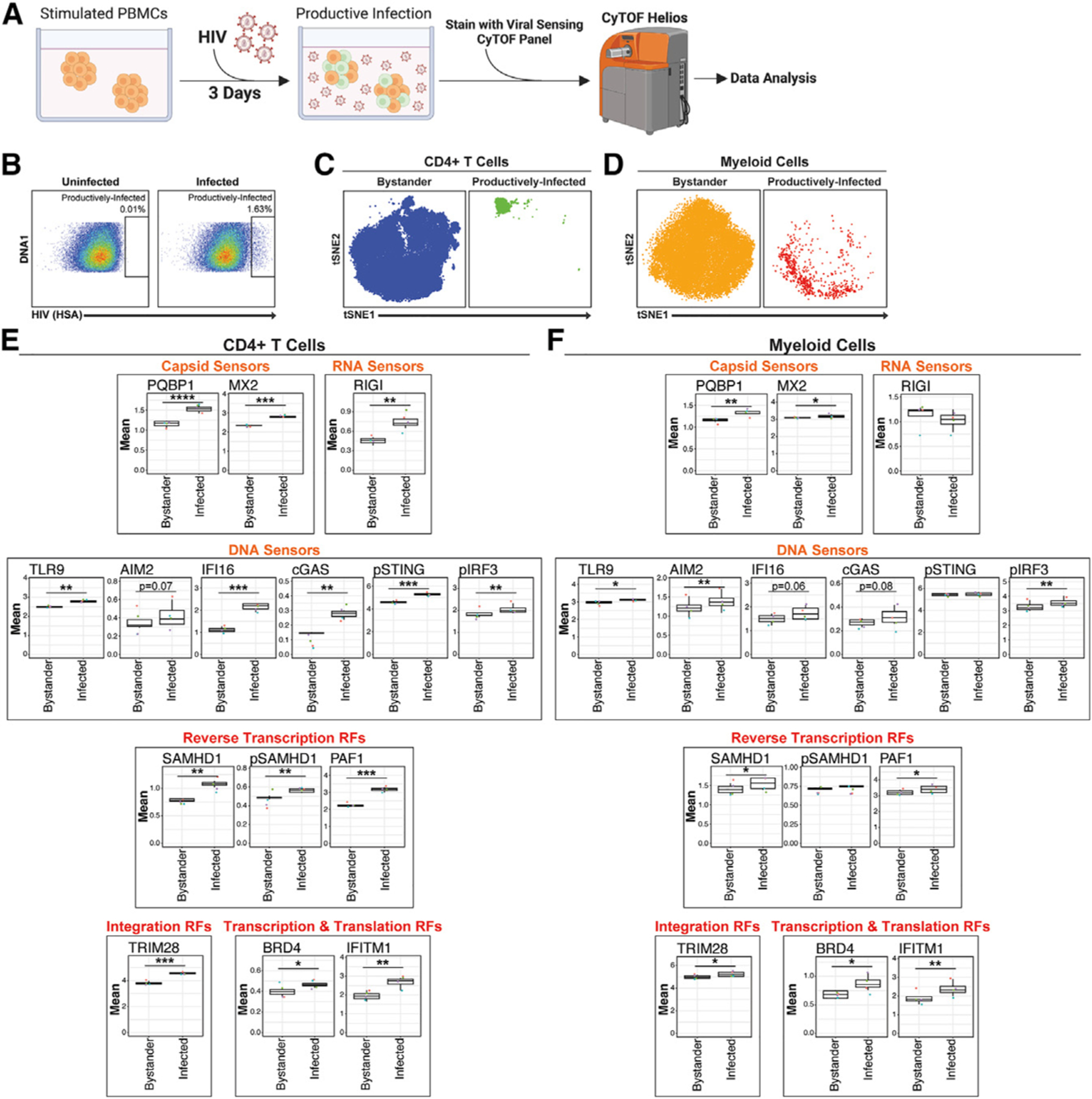
Productively infected cells upregulate viral sensors and restriction factors (A) Productive infection assays experimental design. PHA-stimulated PBMCs (*N* = 4) were mock-treated or exposed to HIV-F4.HSA for 3 days and then analyzed by VISOR-CyTOF. Analyses were performed on events corresponding to bystander or productively infected cells, which were defined based on the expression of long terminal repeat-driven reporter gene HSA. Gating strategies are shown in Figures S5B and S5C. Created with BioRender.com. (B) Identification of productively infected cells by VISOR-CyTOF. Events were pre-gated on live, singlet, CD19^–^CD8^–^cells. (C and D) tSNE plots of bystander and productively infected CD4^+^ T (C) or myeloid (D) cells analyzed by VISOR-CyTOF, highlighting dissimilarity of bystander cells to their productively infected counterparts. tSNE plots were generated using all markers in the CyTOF panel. (E and F) Productively infected cells express high levels of VISORs relative to their bystander counterparts. Shown are viral sensors and restriction factors that are significantly elevated in productively infected CD4^+^ T (E) and myeloid (F) cells relative to their bystander counterparts. Viral sensors significantly upregulated in productively infected CD4^+^ T and myeloid cells were capsid sensors (PQBP1 and MX2) and DNA sensors (TLR9, AIM2, IFI16, cGAS, and pIRF3). In productively infected CD4^+^ T but not myeloid cells, HIV RNA sensors RIGI and pSTING were upregulated. Restriction factors upregulated in both productively infected CD4^+^ T and myeloid cells were those blocking HIV reverse transcription (SAMHD1 and PAF1), nuclear import (MX2), integration (TRIM28), transcription (TRIM28, IFI16, and BRD4), and translation (IFITM1). Productively infected CD4^+^ T but not myeloid cells also upregulated inactive pSAMHD1. *p < 0.05, **p < 0.01, ***p < 0.001, ****p < 0.0001, as assessed using Student’s two-sided paired t tests. Error bars correspond to SD.

To study productively infected cells, we inoculated PHA-stimulated PBMCs with HIV-F4.HSA virus (Figure S5B) and analyzed them 3 days later by VISOR-CyTOF for productively infected (‘‘infected’’) and HIV-exposed but uninfected (‘‘bystander’’) cells (Figures 6A and 6B). As for the fusion assay, cells from a mock-treated culture never exposed to HIV (‘‘uninfected’’) were analyzed in parallel. By tSNE, productively infected CD4^+^ T (Figure 6C) and myeloid (Figure 6D) cells segregated distinctly from their bystander counterparts, suggesting significant viral-induced remodeling, as previously reported.^35,41^ In stark contrast to the fusion data, we found that all VISORs that were differentially expressed between productively infected and bystander CD4^+^ T (Figure 6E) and myeloid cells (Figure 6F) were upregulated in the productively infected ones. For CD4^+^ T cells (Figure 6E), all 15 VISORs analyzed were upregulated upon productive infection. These VISORs included all of those that were downregulated in HIV-fused cells (Figure 5E), in addition to TLR9, pSTING, and pSAMHD1. For myeloid cells, with the exception RIGI and pSTING, all VISORs downregulated in HIV-fused cells (Figure 5F) were upregulated in the productively infected ones (Figure 6F). In addition, we found productive infection to upregulate IFI16 and PAF1 in myeloid cells (Figure 6F). In terms of VISORs commonly upregulated in both CD4^+^ T and myeloid cells upon productive infection, these included sensors of HIV capsid (PQBP1 and MX2) and DNA (TLR9, AIM2, IFI16, cGAS, and pIRF3) and restriction factors targeting reverse transcription (SAMHD1 and PAF1), nuclear import (MX2), integration (TRIM28), transcription (TRIM28, IFI16, and BRD4), and translation (IFITM1) (Figures 6E and 6F). As for the fusion data, similar results were found when comparing the productively infected cells to uninfected (instead of bystander) cells (Figures S7A and S7B), but with some differences not reaching statistical significance (RIGI, AIM2, and pSAMHD1 for CD4^+^ T cells and TLR9 for myeloid cells), likely due to the more heterogeneous nature of the uninfected cell population.

### Upregulating expression of HIV DNA sensors and restriction factors targeting reverse transcription and translation restricts HIV infection

**Figure 7.**
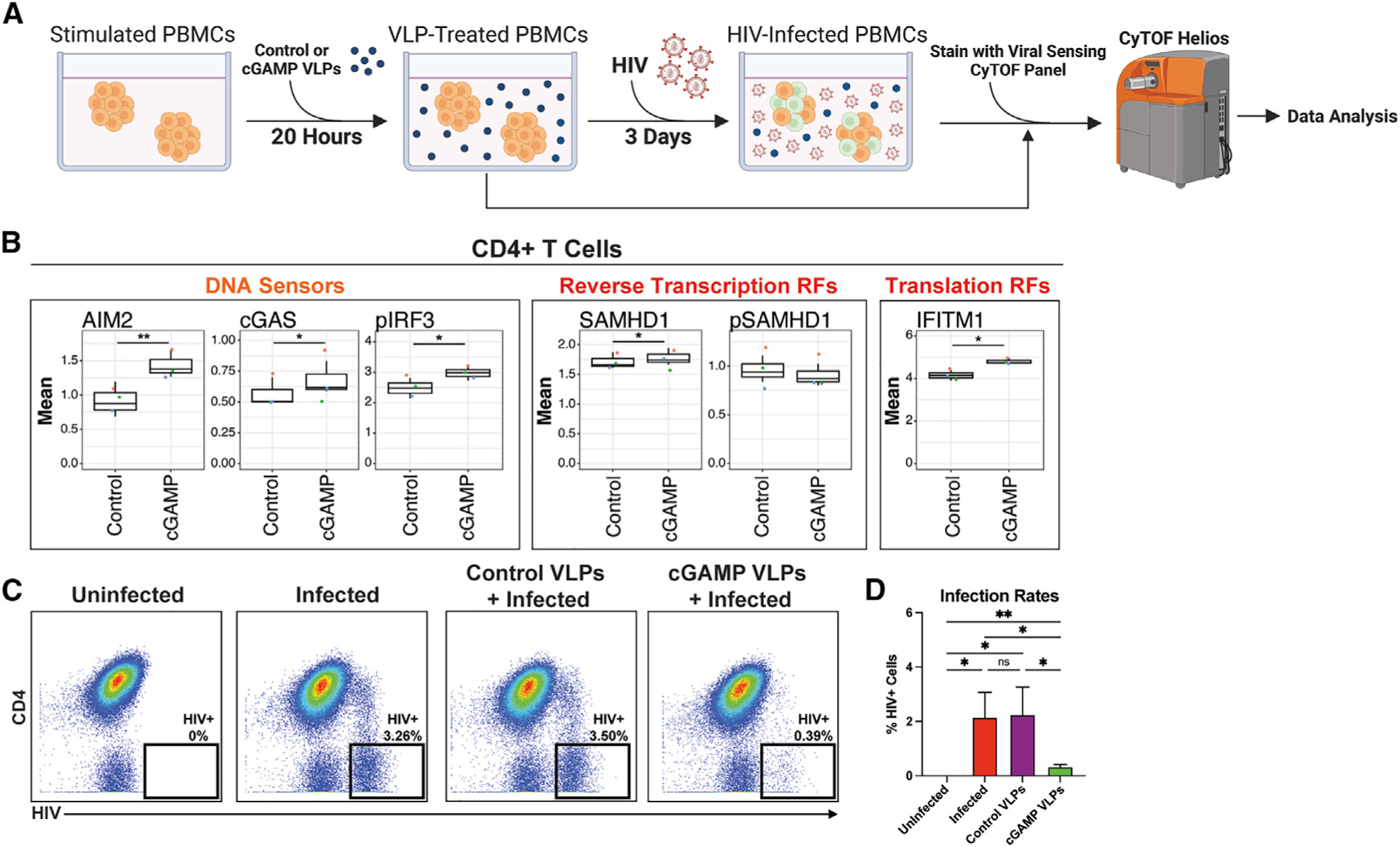
Early elicitation of T1IFN response upregulates DNA sensors and restriction factors leading to inhibition of HIV infection (A) Experimental design for HIV infection of cGAMP-stimulated PBMCs. PHA-stimulated PBMCs (*N* = 3) were incubated for 20 h with cGAMP-containing viral-like particles (VLPs), or control VLPs harboring catalytically inactive cGAS mutant, and then analyzed by VISOR-CyTOF. In parallel, VLP-treated cells from the same donor were mock-infected or exposed to HIV-F4.HSA, cultured for 3 days, and monitored for infection rates. Created with BioRender.com. (B) cGAMP-treated CD4^+^ T cells exhibited elevated expression of HIV DNA sensors (AIM2, cGAS, and pIRF3), as well as restriction factors targeting reverse transcription (SAMHD1) and translation (IFITM1). By contrast, inactive pSAMHD1 trended lower among cGAMP-treated cells. *p < 0.05, **p < 0.01, as assessed using Student’s two-sided paired t tests. (C and D) cGAMP-treated CD4^+^ T cells restrict HIV infection. Representative CyTOF plots of uninfected and infected PBMC cultures treated with control or cGAMP VLPs (C) and cumulative results from 4 independent donors (D). Results were pre-gated on live, singlet, CD19^–^CD3^+^CD8^–^cells. *p < 0.05, **p < 0.01; n.s., non-significant, as assessed using one-way repeated measures ANOVA followed by Tukey’s multiple comparisons test.

Our *in vitro* infection data presented thus far suggest that HIV preferentially fuses to cells with low levels of VISORs, yet productively infected cells harbor high levels of most of these same VISORs, likely the result of a cell-intrinsic futile host response trying to restrict HIV replication. We reasoned that, if VISORs are upregulated earlier, viral restriction may be more effective. To test this, we first assessed the extent to which the various VISORs could be upregulated through eliciting a T1IFN response. To this end, we treated PHA-stimulated PBMCs with 2'3'-cyclic GMP-AMP (cGAMP)-containing viral-like particles (VLPs) (hereafter referred to as cGAMP VLPs) to elicit a T1IFN response^70,71^ followed by VISOR-CyTOF analysis (STAR Methods; Figure 7A). While the numbers of CD4^+^ T cells were unaltered after cGAMP VLP treatment, the numbers of myeloid cells drastically decreased (Figure S8A), presumably due to cGAS-mediated cell death through STING activation, which has been described in monocytes.^72^ We therefore focused these studies on CD4^+^ T cells. Relative to control cells (exposed to VLPs lacking cGAMP), CD4^+^ T cells treated with cGAMP VLPs upregulated expression of multiple HIV DNA sensors (AIM2, cGAS, and pIRF3) (Figures 7B and S8B). They also upregulated restriction factors SAMHD1 (but not inactive pSAMHD1) and IFITM1. By contrast, cGAMP treatment did not affect expression of VISORs associated with HIV capsid and RNA sensing, or those restricting HIV nuclear import, integration, or transcription (Figure S8B). Importantly, upregulation of these VISORs was associated with a significant decrease in the susceptibility of the cGAMP-treated cells to productive infection (Figures 7C and 7D). Collectively, these results demonstrate that cGAMP induction of T1IFN prior to HIV exposure induces expression of sensors of viral DNA and restriction factors targeting reverse transcription and viral translation, which elicits an antiviral state protective against productive infection by HIV.

## DISCUSSION

In this study, we developed a new tool, VISOR-CyTOF, to perform in-depth single-cell characterization of viral sensors and restriction factors in conjunction with immunophenotyping. VISOR-CyTOF provides a first protein-level ‘‘atlas’’ view of VISOR expression across tissue compartments and immune cell subsets. Moreover, by performing *in vitro* fusion and productive infection assays, we identified unique characteristics of HIV-susceptible cells at early and late stages of the HIV replication cycle. Together, our findings highlight the diverse mechanisms by which HIV target cells sense the virus and the varied strategies HIV and host cells use to succeed over the other.

Overall, VISOR expression was highest in myeloid cells, intermediate in T cells, and lowest in B cells. This pattern may reflect the different roles of these immune cells. Myeloid cells, as first responders, may benefit from high expression of VISORs to rapidly detect and respond to microbial pathogens. T cells can directly engage with and lyse infected cells and therefore may benefit from somewhat elevated VISOR expression. In contrast, low expression of VISORs in B cells may serve to keep them in a resting state to avoid triggering an autoimmune antibody response, which can occur when PRRs such as TLRs are activated in conjunction with B cell receptor engagement with cognate antigen.^73^

Within each subset, VISOR expression was also affected by tissue localization. Expression of VISORs was generally higher among immune cells from the gut and lung relative to non-mucosal tissues. Upregulation at mucosal sites may be the consequence of sensing commensal microbes that reside at these sites. For example, secondary messengers produced by commensal bacteria (e.g., 3'3'-*c*-di-AMP or 3'3'-*c*-di-GMP) can lead to upregulation of STING leading to downstream ISG responses,^74^ and indeed we observed elevation of activated STING among myeloid cells from the gut and lung. Of note, the gut is also a major HIV reservoir, harboring a relatively high burden of HIV DNA and RNA within both myeloid and CD4^+^ T cells.^75–78^ As our tissue specimens were all from PWH, persistent sensing of HIV gene products may have also contributed to high VISOR expression that we observed in the gut. This ongoing HIV sensing may also be a driver of the chronic low-level immune activation and inflammation seen in PWH, even in the context of ART suppression.^79,80^ By contrast to cells from the gut and lung, immune cells from the LN, spleen, and liver exhibited relatively low VISOR expression. The LN and spleen are central sites for priming and boosting antigen-specific immune responses, and low VISOR expression at these sites may serve to prevent excess inflammation, which can be detrimental for such adaptive immune responses.

Although LNs harbor a lower burden of the HIV reservoir,^34^ they still harbor a persistent reservoir of infected cells.^81^ LN Tfh, in particular, have been reported to be a preferential subset for persistence of replication-competent HIV.^47,52^ How is it that with low VISOR expression these cells can still be highly susceptible to infection? This may be due to the propensity of these cells to preferentially undergo latent infection, which is supported by our observation that LN Tfh express low levels of TRIM28 (thereby favoring HIV integration) but high levels of BRD4, IFI16, IFIT1, and IFITM1 (thereby blocking HIV gene expression and promoting HIV latency). Consistent with the notion of restriction acting at the post-integration stage to promote latency is the observation that IFITM1 is preferentially overexpressed in resting CD4^+^ T cells latently infected with HIV.^82^ The lack of active HIV gene expression at both the RNA and protein level within Tfh would conceivably allow these cells to persist by minimizing viral gene products that can be sensed by PRRs or antigen-specific CD8^+^ T cells. Furthermore, the preferential localization of Tfh within the ‘‘immune sanctuary’’ site of the B cell follicles, which HIV-specific CD8^+^ T cells rarely frequent, may further shield these cells from elimination.^83,84^ The B cell follicle also has sub-optimal ART drug penetration,^34,85–88^ which can further promote HIV persistence among Tfh by allowing for low-level HIV replication at this site. Together, these mechanisms may be exploited by HIV to persist long-term in LNs of PWH despite ART.

Besides persistence within CD4^+^ T cells, HIV also persists in myeloid cells, particularly microglia of the brain.^89^ Although we did not analyze microglia, we found that myeloid cells from other parts of the human body overall expressed high levels of VISORs, including SAMHD1, a key restriction factor for HIV. Prior studies have reported SAMHD1 expression in a plethora of different human tissues,^10^ but detailed analysis of exactly which cellular subsets expressed the most SAMHD1 was not assessed. Our observation that myeloid cells throughout multiple tissues commonly express SAMHD1 suggests that they would be resistant to HIV infection. Indeed, the early targets of infection by HIV are almost exclusively CD4^+^ T cells, with minimal infection of myeloid cells.^90,91^ Still, we did see variability in SAMHD1 among myeloid cells, in that expression levels were lower in those from the spleen and LN relative to other tissues; this may explain the enhanced susceptibility of LN myeloid cells to HIV infection.^92^ Further supporting the notion of SAMHD1 playing a key role in broad restriction of HIV infection of myeloid cells is our finding that HIV-fused myeloid cells express high levels of SAMHD1 relative to their unfused counterparts. This, together with the relatively low levels of phosphorylated (and inactive) SAMHD1 among HIV-fused myeloid cells, suggests that HIV enters myeloid cells harboring an intracellular environment poorly suited for supporting efficient HIV replication.

By contrast, HIV-fused CD4^+^ T cells expressed *low* levels of SAMHD1 and in fact low levels of most VISORs examined. VISORs weakly expressed among HIV-fused CD4^+^ T cells included sensors of HIV capsid (PQBP1 and MX2), RNA (RIGI), and DNA (AIM2, IFI16, cGAS, and pIRF3), which would render HIV able to enter and reverse transcribe without alerting the cell. Additionally, restriction factors targeting reverse transcription (SAMHD1 and PAF1), nuclear import (MX2), integration (TRIM28), transcription (TRIM28, IFI16, and BRD4), and translation (IFITM1) were also diminished in these cells, which would allow HIV to complete all the major stages of its replication cycle (Figure S1). Why HIV-fused CD4^+^ T cells harbor low levels of VISORs is unclear but does not appear to be associated with differences in HIV receptor/co-receptor expression, which was similar between fused and unfused cells.

In stark contrast to the fusion data, we found that all VISORs examined were upregulated in productively infected CD4^+^ T cells compared to bystander cells. However, this upregulation was not sufficient to block infection as productive infection had already occurred in these cells. It is only when VISORs were upregulated prior to exposure to HIV—through administration of cGAMP VLPs—that we saw potent restriction of HIV infection. The VISORs associated with this restriction included SAMHD1 (with a corresponding downregulation of inactivated pSAMHD1), which presumably shifted the originally permissive CD4^+^ T cell into a refractory state more reminiscent of the HIV-fused myeloid cells. The restriction factor IFITM1 was also upregulated, ensuring that, if any HIV escapes restriction by SAMHD1, it would still be restricted at the protein translation stage. Together, these results demonstrate that early induction of select VISORs can effectively restrict HIV in activated CD4^+^ T cells, while post-fusion upregulation of a broad array of VISORs cannot.

In conclusion, VISOR-CyTOF has provided a detailed map of HIV sensing and restriction across a broad array of immune cells and tissues. Elevated VISOR expression in mucosal tissues, particularly the gut and lung, suggests an adaptation to sensing commensal microbes and possibly persistent HIV, in contrast to the quiescent environment of the LNs and spleen characterized by low VISOR expression. SAMHD1 is expressed in myeloid cells across tissue compartments and in myeloid cells fusogenic for HIV. Conversely, HIV-fused CD4^+^ T cells exhibit low expression of SAMHD1 and all categories of VISORs examined, but upregulation of these factors prior to HIV exposure elicits a restrictive state. Altogether, our study highlights the importance of considering both cellular and tissue-specific contexts when studying the dynamics of HIV infection and persistence.

### Limitations of the study

Our study has limitations. First, our assessment of VISOR expression among tissue cells was limited to PWH, as it leverages the Last Gift cohort of altruistic PWH who wish to contribute to the HIV community by donating their bodies for HIV research. Future efforts should recruit PWOH for comparative studies to determine the role of HIV infection on the observed VISOR expression patterns. Second, because we did not have virological data from each of the analyzed tissues, we could not associate VISOR expression patterns with HIV gene expression. Third, due to limited CyTOF channel availability and our desire to include both VISOR and phenotyping markers, we could not deeply phenotype all immune subsets. For example, we limited our definition of Trm to Tm cells expressing CD69 and did not include other Trm phenotyping markers, such as CD103, CD49a, CD101, and CXCR6,^13,27^ and we did not include classic myeloid cell subsetting markers such as CD16, CD11b, CD11c, CD68, CD163, CD1c, and CD141.^93,94^

## RESOURCE AVAILABILITY

### Lead contact

Further information and requests for resources and reagents should be directed to and will be fulfilled by the lead contact, Nadia R. Roan (nadia.roan@gladstone.ucsf.edu).

### Materials availability

This study did not generate new unique reagents.

### Data and code availability

- The raw datasets generated for this study can be found in the public repository Dryad and accessible at the following link: https://10.5061/dryad.vhhmgqp2q.
- This paper does not report original code. Any additional information required to reanalyze the data reported in this paper is available from the lead contact upon request.

## STAR★METHODS

### EXPERIMENTAL MODEL AND STUDY PARTICIPANT DETAILS

#### Last Gift study participants and tissue collection

The Last Gift Program includes an observational end-of-life cohort of PWH who were diagnosed with a terminal illness and who altruistically donate their bodies for HIV research.^100^ This study is approved by the University of California at San Diego Office of Human Research Protections Program (IRB #160563). Demographical and clinical characteristics of the study participants (*N* = 7) are summarized in Table S3. All participants were at least 18 years of age, provided written informed consent, and had been on combination ART prior to enrollment in Last Gift. Tissue specimens were collected within 8 h of death by rapid research autopsy, and then shipped on ice from University of California at San Diego (UCSD) to Gladstone Institutes/University of California at San Francisco (UCSF) for immediate processing.

### METHOD DETAILS

#### Tissue processing and single-cell isolation

Fresh tissue specimens (spleen, lymph nodes, gastrointestinal tract, male reproductive tract, liver, kidney, bone marrow, heart, and lung) were first dissected into 4 mm^3^ pieces. Spleen and lymph node specimens were mechanically dissociated into single-cell suspensions using 5-mL syringes (Fisher Scientific) and 40-µm strainers (Falcon). Tissue specimens from the gastrointestinal tract, male reproductive tract, liver, kidney, heart, and lung were additionally digested for 2 h with 6.4 mg/mL collagenase type I (Worthington Biochemical Corporation) and 100 U/mL hyaluronidase (Sigma-Aldrich) diluted in RPMI medium supplemented with 1X Antibiotic-Antimycotic Solution (100X; Corning), under gentle rotation. Single-cell suspensions were then run through a Falcon 70-μm cell strainer (Fisher Scientific) and cells were counted. If there were fewer than 3 million live cells per tissue, cells were stained with cisplatin and fixed with paraformaldehyde (PFA), as detailed in the ‘‘Fixation of cells for CyTOF’’ section below. If there were more than 3 million live cells per tissue, cells were then subjected to Lymphoprep density gradient medium to enrich for lymphocytes, per the manufacturer’s protocol (StemCell Technologies), prior to cisplatin treatment and PFA fixation.

#### Isolation and culture of peripheral blood mononuclear cells (PBMCs)

Human peripheral blood mononuclear cells (PBMCs) were harvested from Trima reduction chamber buffy coats (Vitalant Blood Bank, San Francisco, CA, USA) using Lymphoprep density gradient medium (StemCell Technologies), according to the manufacturer’s protocol. Isolated PBMCs were cultured in RPMI-1640 media (Corning) supplemented with 10% of heat-inactivated fetal bovine serum (FBS; VWR) and 1% Penicillin-Streptomycin-Glutamine (Thermofisher Scientific) (referred to as complete RPMI) at a density of 2 × 10^6^ cells/ml. To activate T cells for *in vitro* viral fusion or infection assays, purified PBMCs were stimulated for 24 h with 10 μg/mL PHA (Sigma) and 100 IU/mL recombinant human IL-2 (PeproTech), and then cultured for 48 h in complete RPMI supplemented with 20 IU/mL IL-2 in the absence of PHA. At 72 h, stimulated PBMCs were washed with complete RPMI, passed through a 70-μm cell strainer (Fisher Scientific), and resuspended in complete RPMI containing 20 IU/mL IL-2, for subsequent use in viral fusion or infection assays.

#### HIV fusion assays

BlaM-Vpr-containing HIV virions were generated as previously described.^35,68^ Briefly, 80% confluent HEK293T cells were co-transfected with 60 µg of the F4.HSA HIV-1 proviral DNA construct (referred to as HIV-F4.HSA), 20 μg of pCMV-BlaM-Vpr (Addgene), and 10 μg of pAdVAntage (Promega Corporation) vectors per 175 mm flask in DMEM media (Corning) supplemented with 10% of FBS. F4.HSA encodes a replication-competent, CCR5(R5)-tropic transmitted/founder (T/F) HIV-1 harboring an LTR-driven reporter gene murine heat stable antigen (HSA),^35^ while the pCMV-BlaM-Vpr and pAdVAntage plasmids are used for detection of virion fusion and enhancing translation of the plasmids, respectively. At 16 h post-transfection, media was changed to complete DMEM media, consisting of DMEM supplemented with 10% FBS and 1% Penicillin-Streptomycin-Glutamine (Thermofisher Scientific). At 48 h post-transfection, supernatants containing the HIV virions were filtered through a 0.22-μm filter and concentrated by ultracentrifugation at 20,000 rpm (Beckman Coulter Optima XE-90) for 2 h at 4°C. Viral titers were measured by the LentiXp24Gag Rapid Titer Kit (Takara Bio Inc.). Stimulated PBMCs (5 × 10^7^ cells) were incubated with BlaM-Vpr-containing virions in 15 mL falcon tubes using 0.5 µg of p24Gag in 240 μL, similar to approaches described. After incubating for 2 h at 37°C, PBMCs were washed with CO_2_-independent media (Life Technologies) and loaded with 100 µL of CCF2 loading solution (Life Technologies) for 1 h at room temperature. Cells were then washed once with development media (consisting of 2.5 mM probenecid and 10% FBS in CO_2_-independent media) and incubated for another 6 h. Cells were then stained for 15 min at room temperature with the FACS-based LIVE/DEAD Fixable Red Dead Cell Stain kit (Molecular Probes) at a 1:1000 dilution in FACS buffer, consisting of PBS (Corning) supplemented with 2% of FBS (VWR) and 2 mM EDTA (Life Technologies). After two washes with FACS buffer, HIV-fused cells (identified as those color-shifting from green to blue as a result of cleavage of CCF2 by BlaM) were sorted at 4°C using a FACS AriaII (BD Biosciences) under BSL3 conditions, following the gating strategy presented Figure S5A. For each of the 4 donors analyzed, the total number of sorted cells that supported HIV fusion averaged 6.58 × 10^5^ cells (range 2.86 × 10^5^ to 1.03 × 10^6^ cells). Control cells harboring uncleaved CCF2 (exhibiting green fluorescence) were sorted from a parallel uninfected culture. Sorted cells were treated with cisplatin (Sigma-Aldrich) as a CyTOF live/dead marker and fixed with paraformaldehyde (PFA; Electron Microscopy Services) as detailed in the ‘‘Fixation of cells for CyTOF’’ section further below.

#### Production of cGAMP containing virus-like particles (VLPs)

VLPs containing cGAMP (referred to as cGAMP VLPs) were produced by PEI-mediated co-transfection (Thermofisher Scientific) of HEK293T cells, similar to methods previously described.^70,71^ Briefly, 80% confluent HEK293T cells were co-transfected with plasmids encoding Gag-eGFP, the VSV-G envelope, and murine cGAS (pGag-EGFP, pCMV-VSV-G, and pcDNA3-Flag-mcGAS, respectively) at a 2:1:2 ratio in DMEM media supplemented with 10% of FBS. The plasmids encoding HIV Gag-GFP and VSV-G envelope enables VLP production, while overexpression of cGAS produces cGAMP, which is then incorporated into the nascent VLPs to generate cGAMP VLPs. Control VLPs were used as a negative control, and were produced by co-transfecting with a mutated and catalytically inactive murine cGAS (cGAS AA; pcDNA3-Flag-mcGAS-G198A/S199A) instead of pcDNA3-Flag-mcGAS. At 16 h post-transfection, media was changed to complete DMEM media. At 48 and 72 h post-transfection, HEK293T supernatants containing the cGAMP or control VLPs were filtered through a 0.22-mm filter, underlaid with 20% sucrose, and concentrated by ultracentrifugation at 25,000 rpm (Beckman Coulter Optima XE-90) for 2 h at 4°C. VLPs harvested at 48 and 72 h were combined, resuspended in fresh complete RPMI media, and frozen at –80°C until use.

#### HIV productive infection assays

HIV infection assays were performed similar to methods previously described.^35^ Briefly, HIV-F4.HSA viral stocks were produced by PEI-mediated transfection (Thermofisher Scientific) of HEK293T cells with HIV-F4.HSA proviral DNA expression plasmids. At 16 h post-transfection, cells were replenished with complete DMEM, and after another 24 h, HEK293T supernatants (containing virus) were filtered through a 0.22-μm filter, and concentrated by ultracentrifugation at 25,000 rpm (Beckman Coulter Optima XE-90) for 2 h at 4°C. Viral titers were measured using the LentiXp24Gag Rapid Titer Kit (Takara Bio Inc.). Stimulated PBMCs were mock-treated, or infected with 100–200 ng/mL p24Gag of the HIV-F4.HSA viral stocks, for 72 h at 37°C. Where indicated, stimulated PBMCs were pretreated for 20 h with control or cGAMP VLPs (1 μL of VLPs per 10^6^ cells in 200 μL of complete RPMI media) prior to infection. These doses of VLPs were established through titration experiments with control vs. cGAMP VLPs using approaches similar to those previously described.^101^ At the time of harvest, cells were stained with cisplatin and fixed with PFA, as detailed below.

#### Fixation of cells for CyTOF

Cisplatin staining and fixation of cells for CyTOF analysis was performed as previously described.^18,27,41,102,103^ Briefly, up to 6 million cells were resuspended in 2 mL contaminant-free PBS (Rockland) with 2 mM EDTA (Corning). Cells were then incubated for 1 min with an additional 2 mL of PBS/EDTA supplemented with 12.5 μM cisplatin (Sigma-Aldrich). The cisplatin staining was then immediately quenched with 10 mL CyFACS (contaminant-free PBS [Rockland] supplemented with 0.1% bovine serum albumin [BSA; Sigma-Aldrich] and 0.1% sodium azide [Sigma-Aldrich]). Cells were then fixed for 10 min at room temperature in 2% PFA (Electron Microscopy Sciences) diluted in contaminant-free PBS. After washing the cells in CyFACS, cells were resuspended in 100 µL of CyFACS containing 10% DMSO, and stored at –80°C until analysis by CyTOF.

#### CyTOF staining and data acquisition

Staining of cells for CyTOF was conducted as described previously.^18,27,41,102,103^ To minimize cell loss, multiple cisplatin-treated samples were barcoded and combined using the Cell-ID 20-Plex Pd Barcoding Kit (Standard Biotools) per manufacturer’s protocol. Briefly, fixed samples were thawed, washed twice with Maxpar Barcode Perm Buffer (Standard Biotools), and resuspended in 800 µL of Maxpar Barcode Perm Buffer in Nunc 96 DeepWell polystyrene plates (Thermofisher). Each barcode (10 μL/1–3 million cells) was diluted in 100 μL of Maxpar Barcode Perm Buffer, added to each sample, and the mix incubated for 30 min at room temperature. Barcoded samples were washed once with MaxPar Cell Staining Buffer (Standard Biotools), once with CyFACS, and then combined.

Barcoded cells were aliquoted at a concentration of 6 million cells in 200 μL CyFACS/well into Nunc 96 DeepWell polystyrene plates (Thermofisher). The cells were then blocked for 15 min at 4°C with 100 μL/well of sera from mouse (1.5%; Thermofisher), rat (1.5%; Thermofisher), and human (0.3%; AB serum, Sigma-Aldrich). After 2 washes with CyFACS, cells were incubated for 45 min at 4°C in 100 μL/well containing the cocktail of surface antibodies (Table S2). Cells were then washed 3X with CyFACS, and incubated 4°C overnight in 2% PFA (Electron Microscopy Services) diluted in contaminant-free PBS (Rockland). The next day, cells were permeabilized for 30 min at 4°C by incubation with Fix/Perm buffer (eBioscience). After two washes with Permeabilization Buffer (eBioscience), cells were blocked for 15 min at 4°C in 100 μL/well of 15% mouse and 15% rat sera diluted in Permeabilization Buffer. After washing twice with Permeabilization Buffer, cells were incubated for 45 min at 4°C in 100 μL/well containing the cocktail of intracellular antibodies (Table S2) diluted in Permeabilization Buffer. Cells were then washed with CyFACS and incubated for 20 min at room temperature with 250 nM Cell-ID Intercalator-IR (Standard Biotools). After another two washes with CyFACS, cells were incubated overnight at 4°C in 2% PFA (Electron Microscopy Services) diluted in contaminant-free PBS (Rockland). Immediately prior to sample acquisition, cells were washed sequentially with Maxpar Cell Staining Buffer (Standard Biotools), Maxpar PBS (Standard Biotools), and Maxpar Cell Acquisition Solution (Standard Biotools). For acquisition, cells were then resuspended in a 1:10 dilution of EQ Four Element Calibration Beads (Standard Biotools) in Maxpar Cell Acquisition Solution (Standard Biotools). Cells were injected using a wide-bore (WB) injector on a Helios-upgraded CyTOF2 instrument (Standard Biotools) at the UCSF Parnassus Flow Core Facility. Data were acquired at a slow rate of ~300 events/sec to minimize doublet data acquisition.

### QUANTIFICATION AND STATISTICAL ANALYSIS

#### CyTOF data analyses

##### Data processing and normalization

CyTOF datasets were concatenated, normalized to EQ calibration beads, and de-barcoded using CyTOF software according to the manufacturer’s protocol (Standard Biotools). Cells harboring two different barcodes were removed as doublets during this de-barcoding step. The generated FCS files were renamed using R (Version 4.2.1). FlowJo software (BD Biosciences) was used to generate 2D dot plots and gate for events corresponding to live, singlet cells (Figure S9A). Anchor samples included in each CyTOF run were used to normalize batches of experimental datasets using the application CUHIMSR/CytofBatchAdjust in R.^95^

#### Cell subsetting

Live, singlet events (Figure S9A) exported from the CyTOF datasets were sub-gated into CD19^+^ B cells (CD3^–^CD19^+^ cells; Figure S9B), CD14^+^ myeloid cells (CD3^–^CD19^–^CD14^+^ cells; Figure S9C), CD8^+^ T cells (CD19^–^CD3^+^CD8^+^; Figure S9D), and CD4^+^ T cells (CD19^–^CD3^+^CD8^–^CD4^+^; Figure S9E). T cells were gated into memory (Tm) and naive (Tn) subsets based on expression of the memory marker CD45RO and naive marker CD45RA (Figures S9D and S9E). CD4^+^ Tm cells were further sub-gated into central memory cells (Tcm cells; CD27^+^CCR7^+^), effector memory cells (Tem; CD27^−^CCR7^−^), transitional memory cells (Ttm cells; CD27^+^CCR7^–^; Figure S9F), T follicular helper cells (Tfh; PD1^+^CXCR5^+^; Figure S9G), regulatory T cells (Treg; CD127^–^CD25^+^; Figure S9H), and CD69^+^ Tm cells (CD45RO^+^CD69^+^; Figure S9I) as a proxy for the T resident memory subset. Productively-infected CD4^+^ T cells were identified as CD19^–^CD3^+^CD8^–^CD4^–^cells expressing the reporter gene HSA (Figure S5B) to account for those that have downregulated cell-surface CD4.^104,105^ Productively-infected CD14^+^ myeloid cells were identified CD19^–^CD8^–^CD3^–^CD14^+^ cells expressing HSA (Figure S5C). The average frequency of HIV-fused cells was 2.3% (range 1.33–3.07%) and the average frequency of productively-infected cells was 0.63% (range 0.52–0.78%) of live, singlet cells. A total of 115,674 HIV-fused cells (average = 28,919/donor) and 2,293 productively-infected cells (average 572/donor) were analyzed.

Following subset gating, cell populations were exported as FCS files and imported into R (version 4.2.1). Data were transformed by the inverse hyperbolic function (arcsinh) transformation as follows:

$$arsinh\left(x\right)=\ln\left(x+\sqrt{x^{2}+1}\right)$$

This transformation is used to standardize the diverse range of raw expression level scales for the measured parameters and minimizes the effect of outliers and extreme numbers. tSNE plots were generated using the ‘Rtsne’ (https://github.com/lvdmaaten/bhtsne/) and ‘ggplot2’ (https://ggplot2.tidyverse.org/) packages. The following settings were implemented: iteration = 1000, perplexity = 30, and theta = 0.5. Markers used in the upstream gating strategy and non-cellular markers (e.g., live/dead stain, Pd barcodes) were excluded as tSNE parameters.

#### Cell clustering

FCS files corresponding to total CD4^+^ T cells and myeloid cells were arcsinh transformed in R and exported as csv files for clustering analyses. Biological (tissue type, participant) and technical (batch) variables were visualized using the DimPlot function in the Seurat package.^96^ As batch effects were evident, Harmony^97^ batch correction was performed for each cell type (with the grouping variables set to batch and participant, and the lambda parameter set to 0.01 for both groups) to reduce variabilities introduced by technical batches and inherent inter-individual differences. Specifically, the matrix representing the cell embeddings $\left(U\right)$ generated using Principal Component Analysis (PCA) applied on the centered-scaled arc-sinh transformed marker values matrix $\left(X\right)$ was used as input into the Harmony method:

(Equation 1) $$X=UV^{⊤}$$

where $X\in {\mathbb{R}}^{n\times m}$ represented the centered scaled matrix, $U\in {\mathbb{R}}^{n\times m}$ represented the cell embeddings matrix, $V\in {\mathbb{R}}^{m\times m}$ represented the feature loadings matrix, n represented the numbers of cells, and m represented the number of features/markers. The corrected centered-scaled marker values $\left(X^{cor}\right)$ were then computed as:

(Equation 2) $$X^{cor}=U^{cor}V^{⊤}$$

where $U^{cor}$ represented the corrected cell embeddings matrix output generated by the Harmony method. Batch-corrected principal components were then used for dimensionality reduction using tSNE. The optimal clustering resolution parameters were determined using Random Forests^98^ and a silhouette score-based assessment of clustering validity with subject-wise cross-validation. This procedure is described in greater detail in George et al.^103^ Clustering was performed using Seurat’s FindCluster function with optimal resolution, using all available principal components and the default Louvain clustering algorithm.

#### Statistical analysis

The statistical tests are indicated in the figure legends, with *p < 0.05, **p < 0.01, ***p < 0.001, ****p < 0.0001, and n.s. = non-significant *p*-values. Briefly, for mean signal intensity (MSI) analyses, raw expression values of selected markers from each cell were arcsinh transformed in R (version 4.2.1). Student’s two-sided paired (by donor) t-tests were used to test for differences in MSI of each parameter between cell populations and *p*-values were adjusted for multiple testing using the Holm-Sidak method, where applicable. This was implemented with the ‘stats’ R base package. For quantification of the effects of cGAMP-treatment on HIV infection, a one-way repeated measures ANOVA was performed and then *p*-values were adjusted for multiple testing using the Tukey’s method for multiple comparisons (GraphPad Prism, version 10.2.0). For cluster membership associations, a generalized linear mixed model (GLMM, implemented in the lme4^99^ package in R with family argument set to the binomial probability distribution) with a random effect for sample was used to estimate the association between cluster and tissue type. Cluster membership was defined as the ratio of all the cells from a given sample in the cluster under consideration divided by all other cells from that sample. The change in cluster membership between tissue types was estimated as a log odds ratio, defined as the change in the log odds of cluster membership between tissue types. This was estimated with the ‘emmeans’ R package (https://github.com/rvlenth/emmeans/) using the GLMM model fit. The two-sided *p*-values corresponding to the null hypothesis of an odds ratio value of 1 were computed based on a Z-statistic in the GLMM model fit. These *p*-values were corrected for multiple testing across all pairwise tissue comparisons using the Benjamini-Hochberg method. All error bars correspond to standard deviation (SD). Graphs were generated using R (version 4.2.1) or GraphPad Prism (version 10.2.0), respectively.

## Supplementary Material

1

## Figures and Tables

**Table:** KEY RESOURCES TABLE

REAGENT or RESOURCE	SOURCE	IDENTIFIER
Antibodies		
CD57	Biolegend	Cat#359602; RRID:AB_2562403
HLADR	Thermo Fisher Scientific	Cat#Q22158; RRID:AB_2556514
CD25	BD Biosciences	Cat#555430; RRID:AB_395824
CD19	Standard BioTools	Cat#3142001B; RRID:AB_3661857
IFI16	Santa Cruz Biotechnology	Cat#sc-8023; RRID:AB_627775
CCR5	Standard BioTools	Cat#3144007A; RRID:AB_2892770
IFITM1	Proteintech	Cat#60074-1-Ig; RRID:AB_2233405
CD8	Standard BioTools	Cat#3146001B; RRID:AB_3661846
cGAS	Cell Signaling Technology	Cat#79978S; RRID:AB_2905508
IFIT3	Novus	Cat#NBP2-71006; RRID:AB_3094693
AIM2	Santa Cruz Biotechnology	Cat#sc-293174; RRID:AB_3665129
HSA (CD24)	Standard BioTools	Cat#3150009B; RRID:AB_2916042
LAG3	Standard BioTools	Cat#3150030B; RRID:AB_3661851
pSTING	Cell Signaling Technology	Cat#40818; RRID:AB_2799187
SLFN11	Santa Cruz Biotechnology	Cat#sc-374339; RRID:AB_10989536
MX2	Santa Cruz Biotechnology	Cat#sc-271527; RRID:AB_10649506
IFIT1	Novus	Cat#NBP2-71005; RRID:AB_3363102
pIRF3	Cell Signaling Technology	Cat#29047; RRID:AB_2773013
SAMHD1	Proteintech	Cat#12586-1-AP; RRID:AB_2183496
TRIM28	R and D Systems	Cat#MAB7785; RRID:AB_3096992
PAF1	Santa Cruz Biotechnology	Cat#sc-514491; RRID:AB_3665128
CCR7	Standard BioTools	Cat#3159003A; RRID:AB_2938859
MX1	Cell Signaling Technology	Cat#62815; RRID:AB_3665127
CD45RO	Biolegend	Cat#304239; RRID:AB_2563752
CD69	Standard BioTools	Cat#3162001B; RRID:AB_3096016
TLR9	Novus	Cat#NBP2-24729; RRID:AB_3272891
CXCR5	Standard BioTools	Cat#3164029B; RRID:AB_3665126
pSAMHD1	Sigma-Aldrich	Cat#MABF934; RRID:AB_3665125
PQBP1	Santa Cruz Biotechnology	Cat#sc-376039; RRID:AB_10989350
CD27	Standard BioTools	Cat#3167002B; RRID:AB_3094744
PD1	BD Biosciences	Cat#562138; RRID:AB_10897007
CD45RA	Standard BioTools	Cat#3169008B; RRID:AB_3665124
CD3	Standard BioTools	Cat#3170001B; RRID:AB_2811085
RIGI	Novus	Cat#NBP2-61849; RRID:AB_3351257
CD38	Standard BioTools	Cat#3172007B; RRID:AB_2756288
BRD4	Abcam	Cat#ab182446; RRID:AB_3665123
CD4	Standard BioTools	Cat#3174004B; RRID:AB_3661864
CD14	Biolegend	Cat#301843; RRID:AB_2562813
CD127	Standard BioTools	Cat#3176004B; RRID:AB_3665122
TIGIT	Standard BioTools	Cat#3209013B; RRID:AB_2905649
Bacterial and virus strains		
HIV-F4.HSA (NL-HSA.6ATRi-C.109FPB4.ecto)	Cavrois et al.^34^	N/A
HIV-F4.HSA virions containing BlaM-Vpr	Cavrois et al.^34,67^	N/A
Biological samples		
Tissues from people with HIV (Last Gift Study)	https://lastgift.ucsd.edu/	N/A
PBMCs from people without HIV	https://www.vitalant.org	N/A
Chemicals, peptides, and recombinant proteins		
16% Paraformaldehyde	Electron Microscopy Sciences	Cat#15710
Fetal Bovine Serum	VWR	Cat#97068-085
Metal Contaminant-Free PBS	Rockland	Cat#MB-008
Normal Mouse Serum	Thermo Fisher Scientific	Cat#10410
Normal Rat Serum	Thermo Fisher Scientific	Cat#10710C
Human Serum from Male AB Plasma	Sigma-Aldrich	Cat#H4522
Iridium Interchelator Solution	Standard Biotools	Cat#201192B
EQ Four Element Calibration Beads	Standard Biotools	Cat#201078
Opti-MEM	Gibco	Cat#31985062
Polyethylenimine HCL (PEI)	Polysciences	Cat#24765
CO_2_-independent media	Life Technologies	Cat#18045-088
CCF2-AM substrate and loading solutions	Life Technologies	Cat#K1032
Probenecid	Sigma-Aldrich	Cat#P8761
BD CompBeads	BD Biosciences	Cat#552843
Collagenase type I	Worthington Biochemical Corporation	Cat#LS004196
Hyaluronidase	Sigma-Aldrich	Cat#H2251
RPMI-1640 medium	Corning	Cat#10-040-CM
Lymphoprep density gradient medium	StemCell Technologies	Cat#07851
Penicillin-Streptomycin-Glutamine	Thermo Fisher Scientific	Cat#10378016
Lectin from Phaseolus vulgaris (Phytohemagglutinin (PHA))	Sigma-Aldrich	Cat#L1668-5MG
Recombinant human IL-2	PeproTech	Cat#200-02-100UG
Antibiotic-Antimycotic Solution, 100X	Corning	Cat#30-004-CI
DMEM (Dulbecco’s Modified Eagle’s Medium)	Corning	Cat#10-013-CV
Critical commercial assays		
MaxPar X8 Antibody Labeling Kit	Standard Biotools	Cat#201169B
Cell-ID 20-Plex Pd Barcoding Kit	Standard Biotools	Cat#201060
Foxp3 Transcription Factor Staining Buffer Set	eBioscience	Cat#00-5523-00
MaxPar Cell Acquisition Solution	Standard Biotools	Cat#201240
MaxPar PBS	Standard Biotools	Cat#201058
MaxPar Cell Staining Buffer	Standard Biotools	Cat#201068
LentiX p24Gag Rapid Titer Kit	Takara Bio Inc	Cat#632200
LIVE/DEAD Fixable Red Dead Cell Stain kit	Thermo Fisher Scientific	Cat#L23102
Deposited data		
Raw CyTOF datasets	This paper	https://10.5061/dryad.vhhmgqp2q
Recombinant DNA		
pAdVAntage	Promega	Cat#E1711
pCMV4-BlaM-Vpr	Addgene	Cat#21950; RRID:Addgene_21950
pHIV-F4.HSA (pNL-HSA.6ATRi-C.109FPB4.ecto)	Cavrois et al.^34^	N/A
pCMV-VSV-G	Addgen	Cat#8454; RRID:Addgene_8454
pGag-EGFP	NIH AIDS Reagent Program	Cat#11468
pcDNA3-Flag-mcGAS	Chauveau et al.^69^; Bridgeman et al.^70^	N/A
pcDNA3-Flag-mcGAS-G198A/S199A	Chauveau et al.^69^; Bridgeman et al.^70^	N/A
Software and algorithms		
FlowJo software (Version 10.10.0)	https://www.flowjo.com/	RRID:SCR_008520
GraphPad Prism (Version 10.2.0)	http://www.graphpad.com/	RRID:SCR_002798
RStudio (2022.07.2 + 576 "Spotted Wakerobin" Release)	https://rstudio.com/	RRID:SCR_000432
R Project for Statistical Computing (Version 4.2.1)	http://www.r-project.org/	RRID:SCR_001905
CUHIMSR/CytofBatchAdjust	Schuyler et al.^95^; https://github.com/CUHIMSR/CytofBatchAdjust	N/A
ggplot2	https://cran.r-project.org/web/packages/ggplot2/index.html	RRID:SCR_014601
Rtsne	https://github.com/jkrijthe/Rtsne	RRID:SCR_016342
Seurat	Hao et al.^96^; https://satijalab.org/seurat/get_started.html	RRID:SCR_016341
Harmony	Korsunsky et al.^97^;https://github.com/immunogenomics/harmony	RRID:SCR_022206
RandomForest Package in R	Breiman^98^; https://cran.r-project.org/web/packages/randomForest/	RRID:SCR_015718
lme4	Bates et al.^99^; https://cran.r-project.org/web/packages/lme4/index.html	RRID:SCR_015654
emmeans	https://CRAN.R-project.org/package=emmeans	RRID:SCR_018734

## Highlights

- Immune subsets differ in viral sensor and restriction factor profiles across tissues
- SAMHD1 is broadly expressed by tissue and HIV-fused myeloid cells
- Tcm, Tem, Trm, Treg, Tfh, and other CD4^+^ T cell subsets differentially express VISORs
- Pre-infection increase of CD4^+^ T cell sensors restricts productive HIV infection
