# Supplementary material for: Anatomical, subset, and HIV-dependent expression of viral sensors and restriction factors
Source: Cell Rep. Author manuscript; Available in PMC 2025 Feb 15. (PMC11829653; doi:10.1016/j.celrep.2024.115202)
Supplement: 1 [file NIHMS2052771-supplement-1.pdf]

Cell Reports, Volume *44*

Supplemental information

Anatomical, subset, and HIV-dependent expression of viral sensors and restriction factors

Ashley F. George, Jason Neidleman, Xiaoyu Luo, Julie Frouard, Natalie Elphick, Kailin Yin, Kyrlia C. Young, Tongcui Ma, Alicer K. Andrew, Ifeanyi J. Ezeonwumelu, Jesper G. Pedersen, Antoine Chaillon, Magali Porrachia, Brendon Woodworth, Martin R. Jakobsen, Reuben Thomas, Davey M. Smith, Sara Gianella, and Nadia R. Roan

SUPPLEMENTAL FIGURES

Figure S1

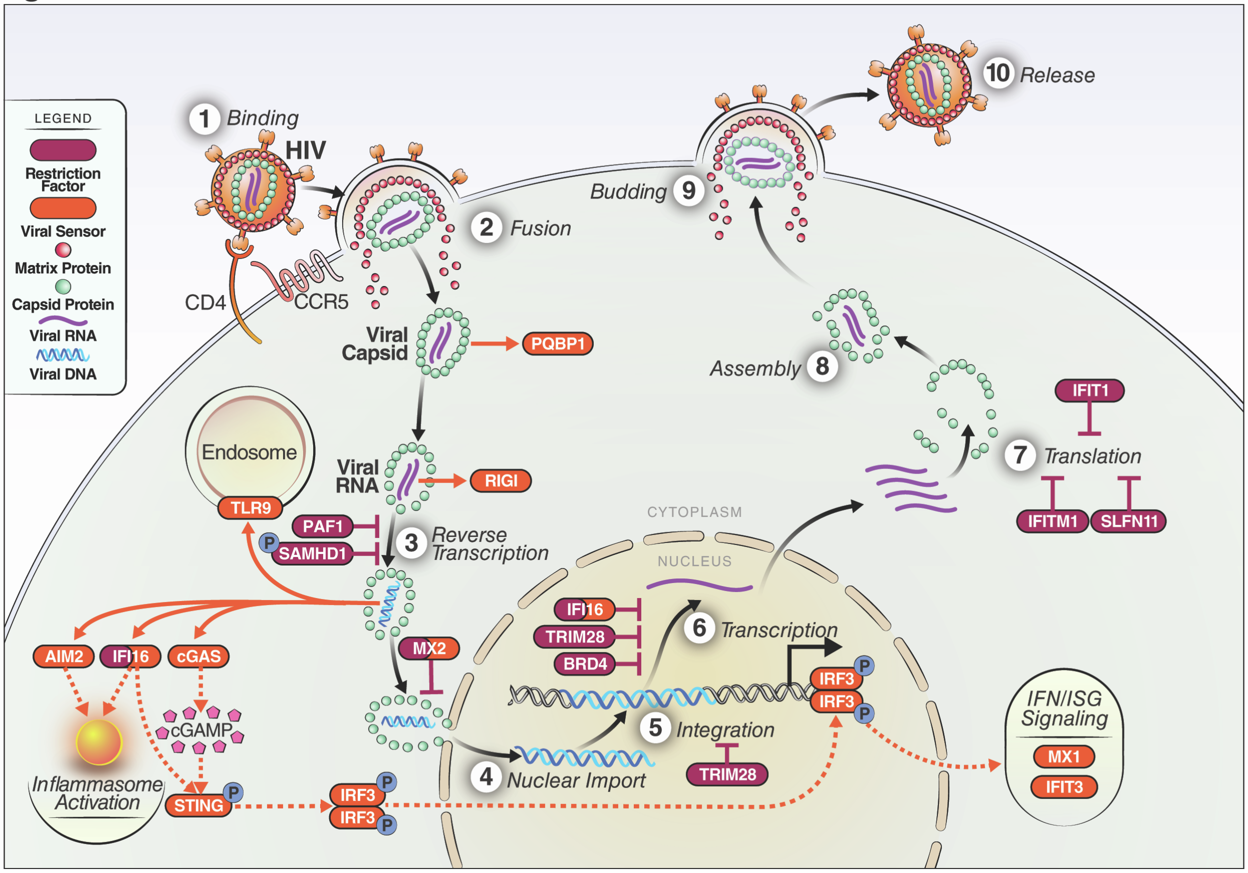

Figure S1. Viral sensors and restriction factors analyzed by VISOR-CyTOF.
Illustrated are the VISORs analyzed in this study in the context of the HIV replication cycle. Solid black arrows depict steps of the HIV replication cycle. VISORs classified as viral sensors are depicted in orange, those classified as restriction factors in red, and those classified as both in both colors. Sensors in the panel include those that can directly sense viral gene products, as well as those that act indirectly, including interferon stimulated genes (ISGs), by propagating initial sensing of the virus. Solid orange arrows denote the viral products recognized by the sensors. Dotted orange arrows highlight signal transduction pathways downstream of viral sensing. Red inhibitory symbols depict the steps at which specific restriction factors can block HIV replication. Of note, our panel did not include late-stage restriction factors that diminish virion release or infectivity of HIV virions released from HIV-infected cells [1], as our study focuses on cell-intrinsic restriction of HIV. **Abbreviations:** VISORs: **VI**ral **S**en**O**r and **R**estriction factors

Figure S2

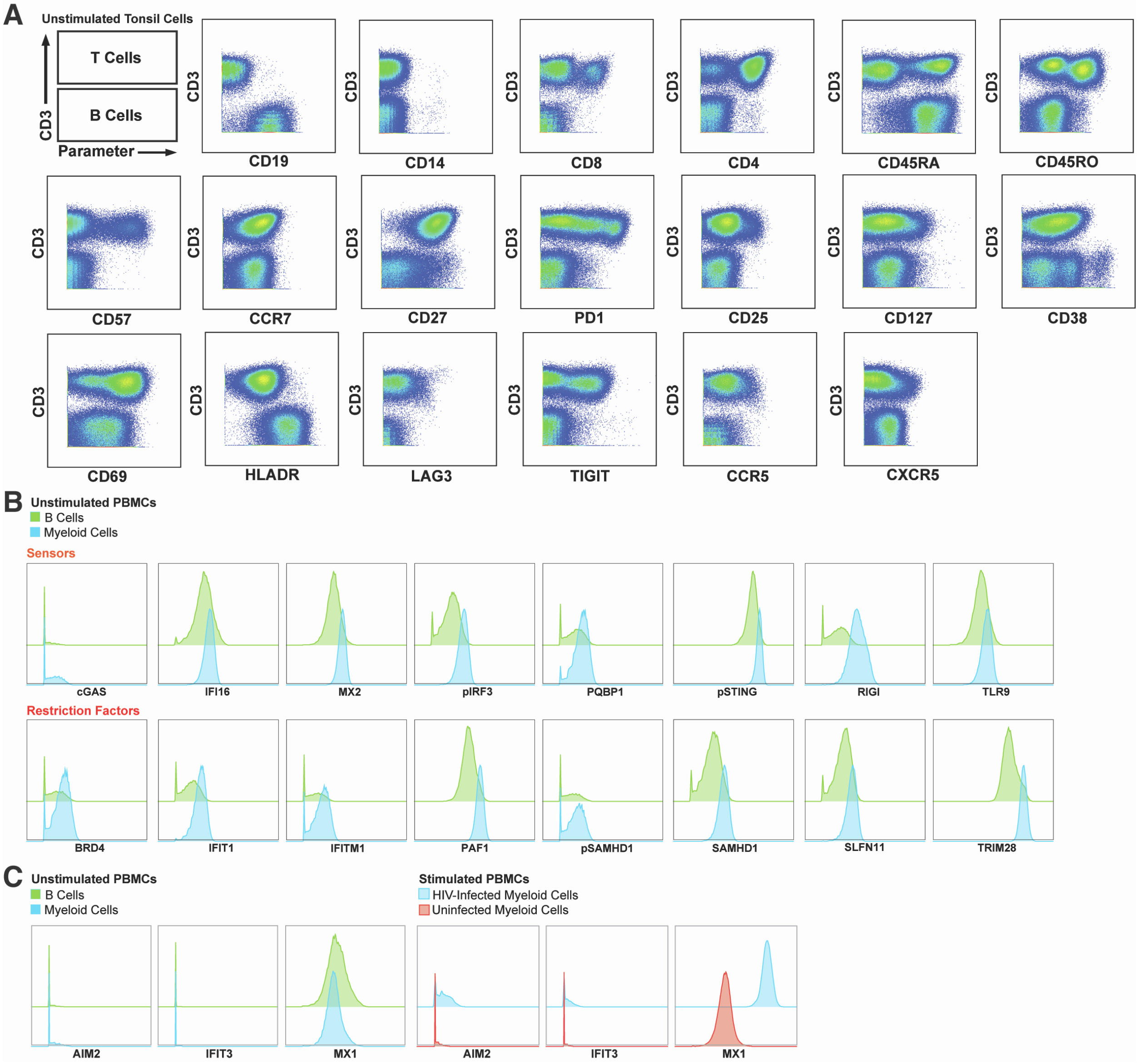

Figure S2. Validation of the VISOR-CyTOF panel.

(A) Validation of phenotyping antibodies. Tonsils, which harbor robust B and T cell populations, were used for phenotyping antibody validation. Total, live singlet events were visualized as 2D plots depicting CD3 on the y-axis and other phenotyping markers on the x-axis. As almost all tonsillar $CD3^-$ cells are $CD19^+$, visualizing CD3 on the y-axis distinguishes T cells (*top half*) from B cells (*bottom half*). The observed differential expression patterns of phenotyping markers among T vs. B cells are similar to those previously reported [2]. **(B)** Validation of VISOR antibodies. Differential expression patterns of VISORs among B and myeloid cell populations from PBMCs were used for VISOR antibody validation. VISOR-CyTOF analysis reveals elevated expression of viral sensors (cGAS, IFI16, MX2, pIRF3, PQBP1, pSTING, RIGI, and TLR9) and restriction factors (BRD4, IFIT1, IFITM1, PAF1, pSAMHD1, SAMHD1, SLFN11, and TRIM28) on myeloid as compared to B cells, consistent with prior reports [3-5]. **(C)** Validation of AIM2, IFIT3, and MX1 VISOR antibodies. While AIM2, IFIT3, and MX1 were not differentially expressed on myeloid as compared to B cells, their expression is elevated after HIV infection of PBMC-derived myeloid cells, consistent with prior reports [6,7].

Figure S3

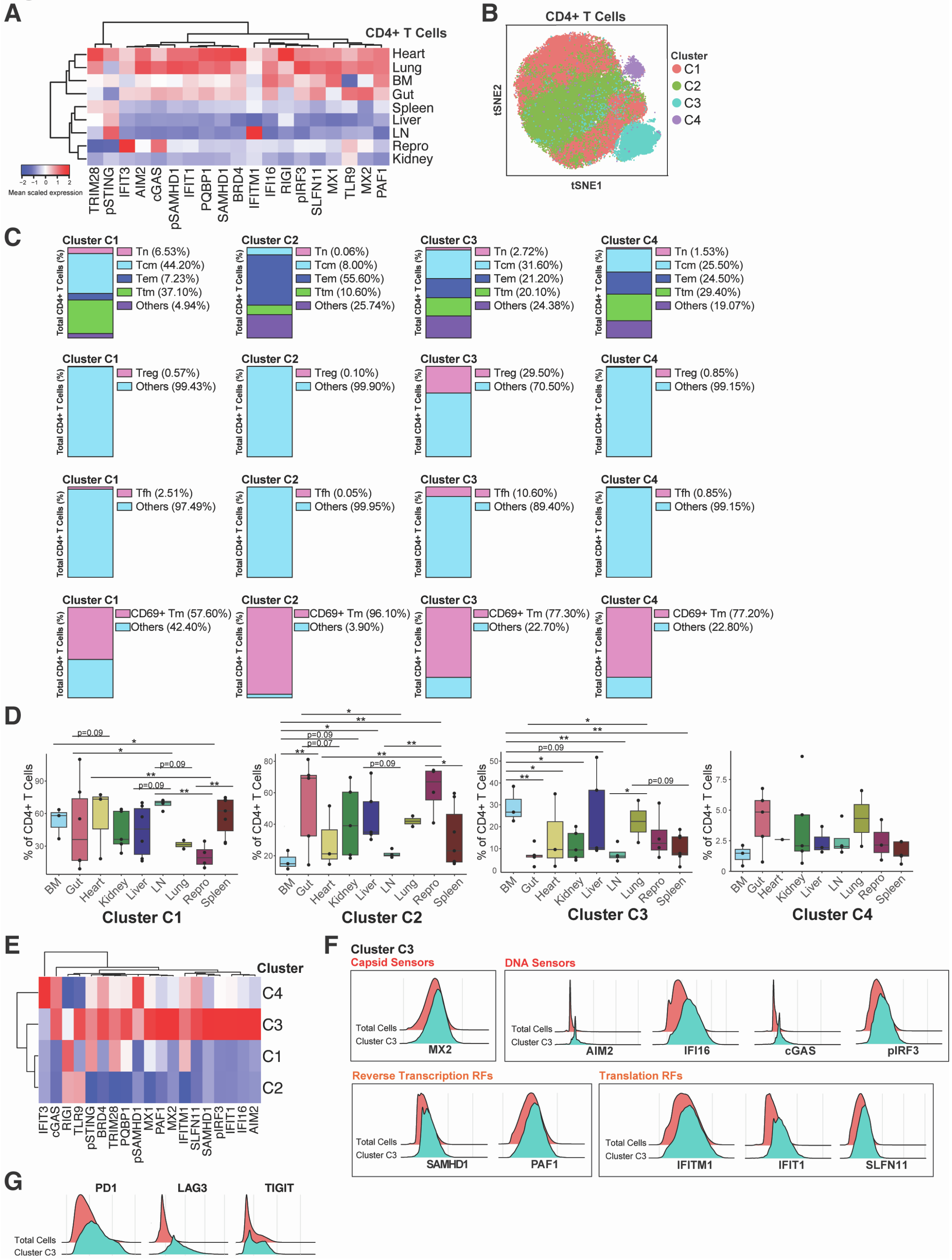

Figure S3. A subset of checkpoint-expressing CD4⁺ T cells enriched in BM and lung expresses high levels of sensors of HIV capsid and DNA, and restriction factors targeting all stages of the HIV replication cycle except for integration.

(A) VISORs are most highly expressed in CD4^{+} T cells from the heart, lung, BM, and gut and most lowly expressed in those from kidney, male reproductive organs, LN, liver, and spleen. **(B)** Louvain clustering identifies four clusters of tissue CD4+ T cells (C1-C4). tSNE plots were generated using all markers in the CyTOF panel. **(C)** Frequencies of Tn, Tcm, Tem, Ttm, Treg, Tfh, and CD69+ Tm among clusters C1-C4. **(D)** Distributions of CD4+ T cell clusters across tissue sites. Cluster C1 cells are enriched in the heart, BM, LN, and spleen; cluster C2 cells are enriched in the gut, male reproductive tract, liver, and kidney; cluster C3 cells are enriched in BM and lung; cluster C4 cells trend highest in the gut and lung. Individual points represent the % of all cells from a given participant that belong to the respective cluster. *$p < 0.05$, **$p < 0.01$, as assessed using GLMM with multiple correction by Benjamini-Hochberg procedure for FDR (Methods). **(E)** VISORs are most highly expressed in cluster C3 cells. **(F)** Cluster C3 cells express high levels of capsid sensor MX2 and the DNA sensors AIM2, IFI16, cGAS, and pIRF3. They also express high levels of restriction factors targeting reverse transcription (SAMHD1 and PAF1), nuclear import (MX2), transcription (IFI16), and translation (IFITM1, IFIT1, and SLFN11). **(G)** Cluster C3 cells express high levels of the checkpoint molecules PD1, LAG3, and TIGIT. Heatmaps denote the column-normalized mean scaled expression of each indicated VISOR within each cluster or tissue. Histograms show expression levels of the indicated marker for the indicated cluster. All error bars correspond to SD.

Figure S4

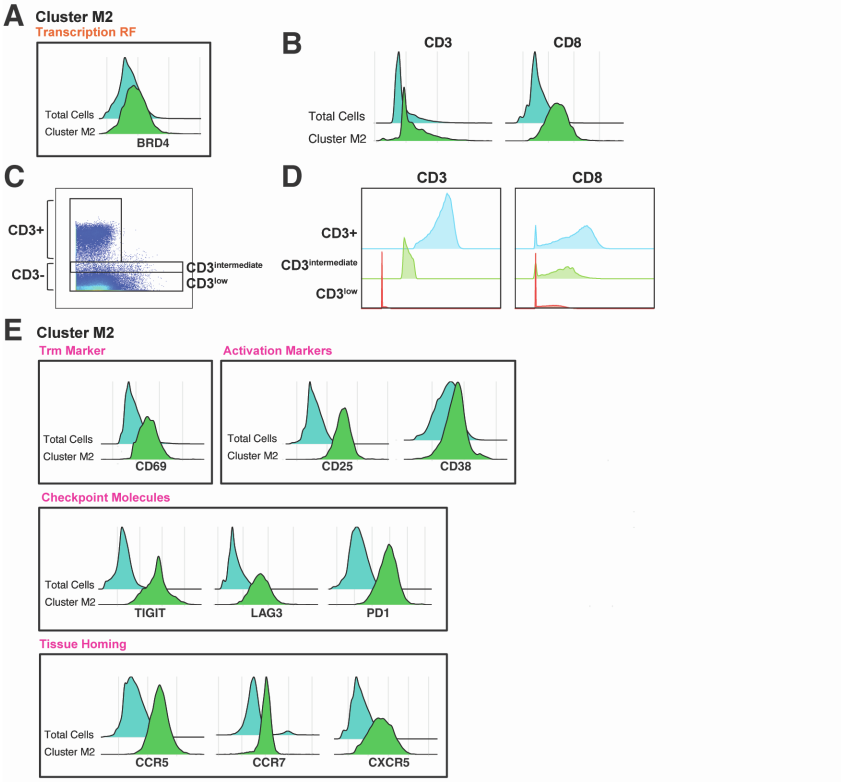

Figure S4. Cluster M2 cells are activated $CD14^{+}CD8^{+}$ T cells expressing BRD4.
(A) Cluster M2 cells express high levels of BRD4. **(B)** Cluster M2 cells express T cell markers CD3 and CD8. **(C)** Representative gating strategy to distinguish $CD3^{+}$ from $CD3^{-}$ cells, which were used to define $CD3^{-}CD14^{+}$ myeloid cells (*left*). CD3- cells were further gated into two sub-populations: $CD3^{intermediate}$ and $CD3^{low}$ (*right*). **(D)** Histograms showing expression of CD3 (*left*) and CD8 (*right*) of cells expressing the three different levels of CD3. Results in panels *C* and *D* are gated on live, singlet cells. **(E)** Cluster M2 cells preferentially express Trm, T cell activation, immune checkpoint, and tissue homing markers suggesting their identity to be CD8+ T cells that that have acquired CD14 [8].

Figure S5

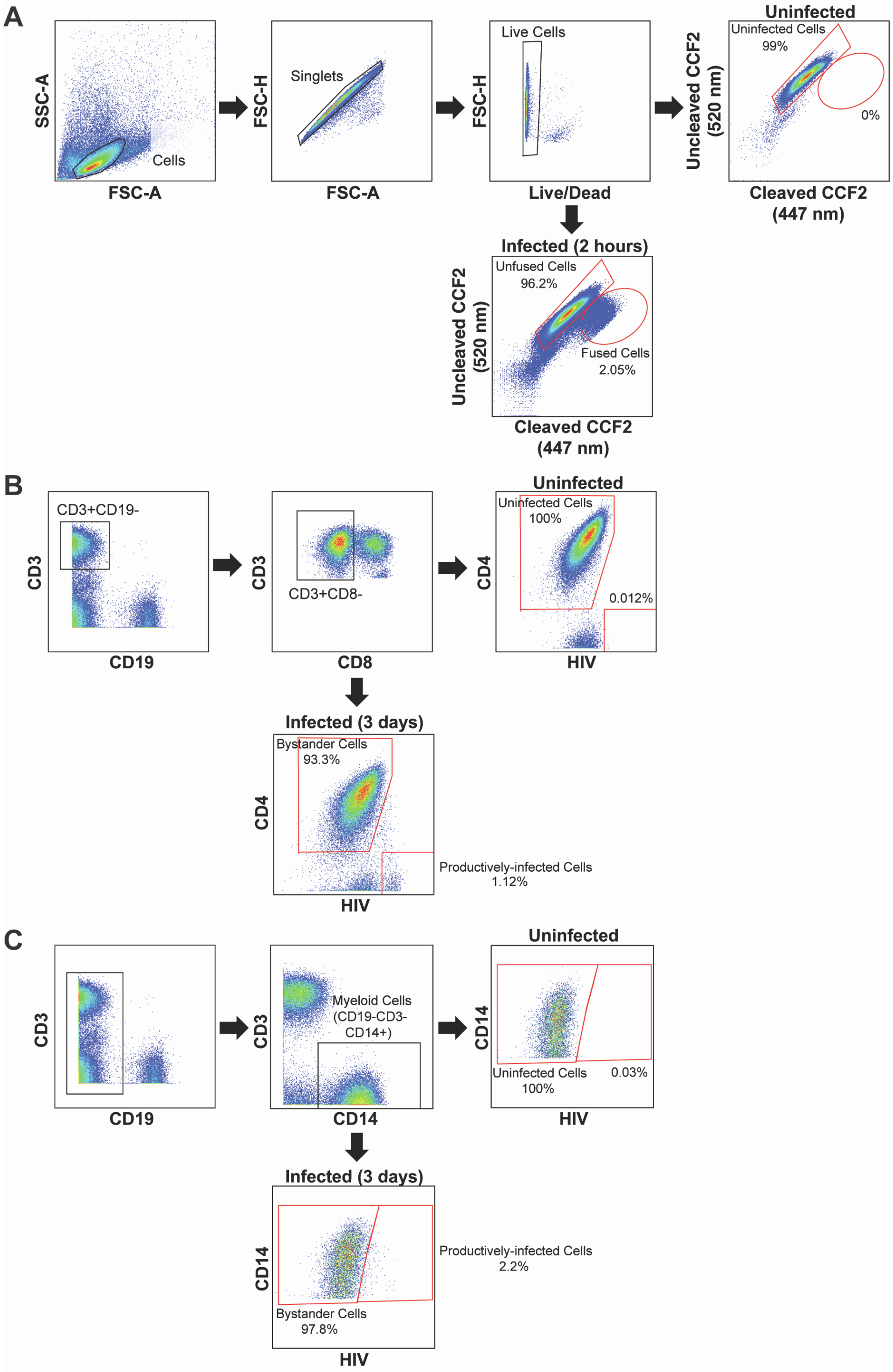

Figure S5. Gating strategies to identify HIV-fused and productively-infected cells.
(A) Representative gating strategy used to identify live, singlet, HIV-fused cells. The gate for HIV-fused cells was established by comparing the HIV-exposed and uninfected samples. Uninfected, fused, and unfused cells were sorted using the depicted gates and were analyzed by VISOR-CyTOF. **(B, C)** Representative gating strategies used for CyTOF analysis of CD4^{+} T (***B***) and myeloid (***C***) cells that were uninfected, productively-infected, or bystander cells. Productively-infected CD4+ T cells were defined as those that had down-regulated cell-surface CD4. All events were pre-gated on live, singlet cells.

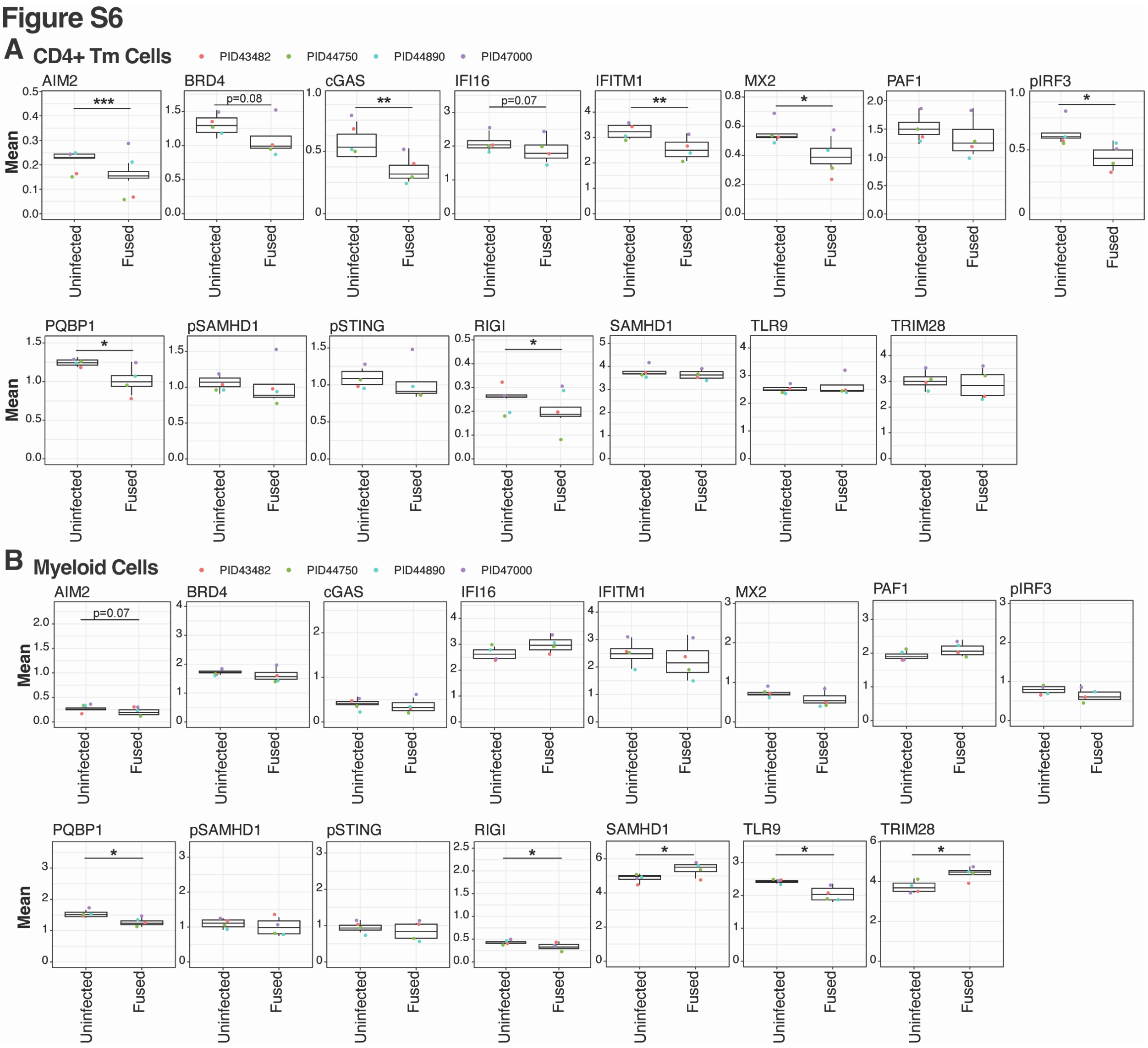

Figure S6. Comparison of mean expression levels of VISORs in uninfected vs. HIV-fused CD4$^+$ Tm and myeloid cells.

Mean expression levels of all VISORs in uninfected vs. HIV-fused CD4$^+$ Tm **(A)** and myeloid **(B)** cells. VISORs are listed in alphabetical order. *$p < 0.05$, **$p < 0.01$, ***$p < 0.001$, ****$p < 0.0001$, as assessed by Student's two-sided paired t-tests. Error bars correspond to SD.

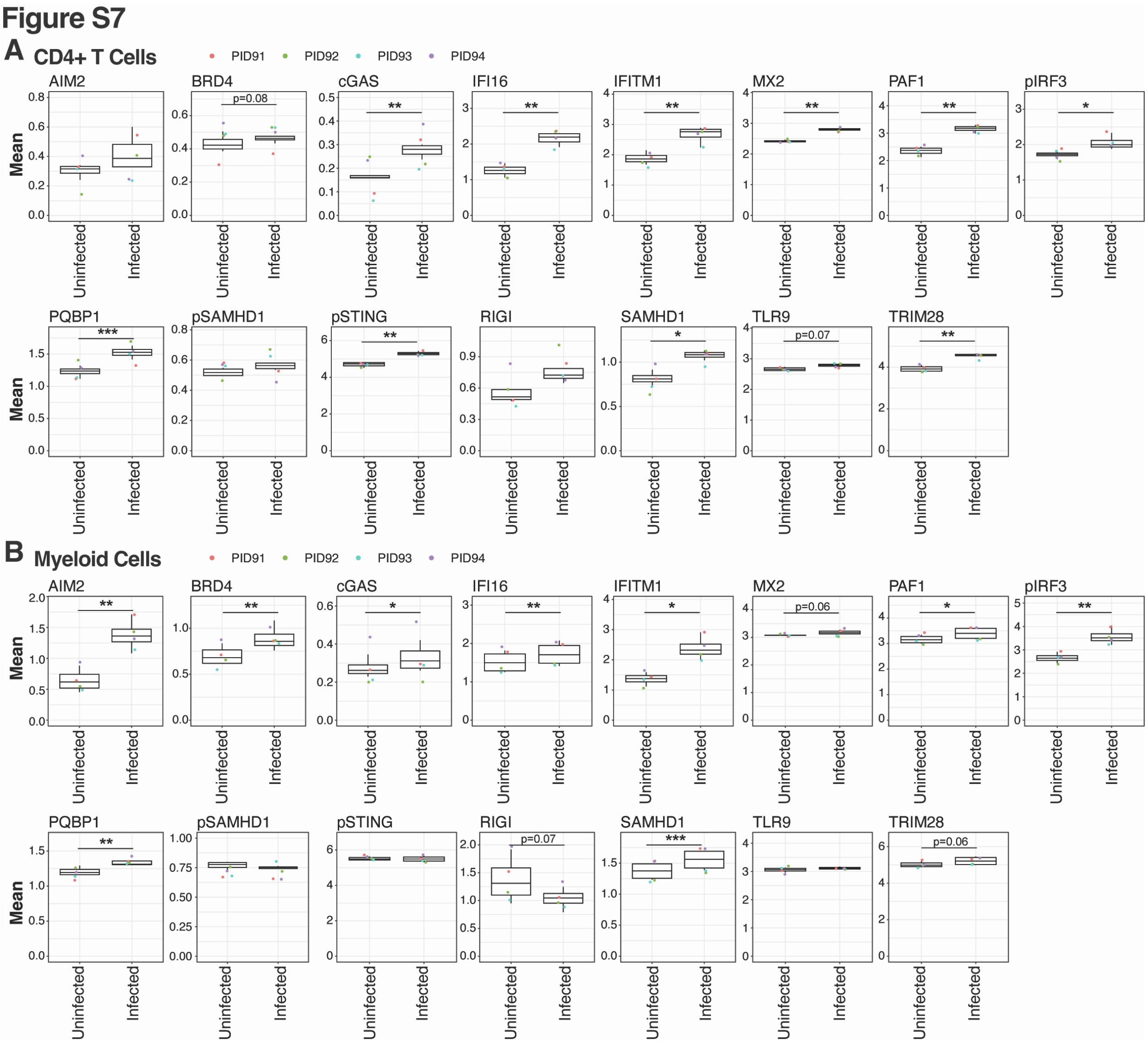

Figure S7. Comparison of mean expression levels of VISORs in uninfected vs. productively-infected CD4+ T and myeloid cells.

Mean expression levels of all VISORs in uninfected vs. productively-infected CD4+ T **(A)** and myeloid **(B)** cells. VISORs are listed in alphabetical order. *p < 0.05, **p < 0.01, ***p < 0.001, ****p < 0.0001, as assessed by Student's two-sided paired t-tests. Error bars correspond to SD.

Figure S8

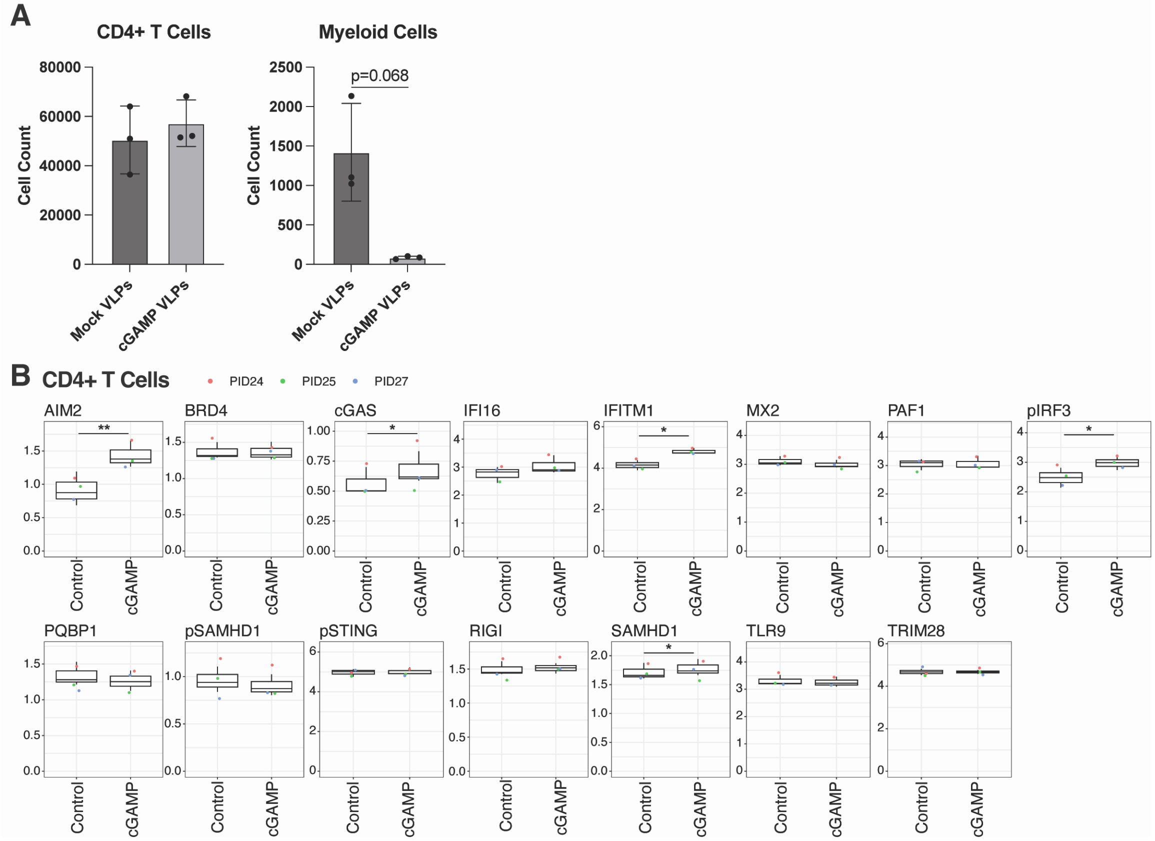

Figure S8. Mean expression levels of VISORs in control and cGAMP-treated CD4+ Tm cells. (A) cGAMP-treatment does not affect total numbers of CD4+ T cells (*left*), but diminishes total numbers of myeloid cells (*right*). **(B)** Mean expression levels of all VISORs in control and cGAMP-treated CD4+ Tm cells. $*p < 0.05$, $**p < 0.01$, $***p < 0.001$, $****p < 0.0001$, as assessed by Student's two-sided paired t-tests. Error bars correspond to SD. VISORs are listed in alphabetical order, and ones differentially expressed in cGAMP-treated cells are shown grouped by category in Fig. 7B.

Figure S9

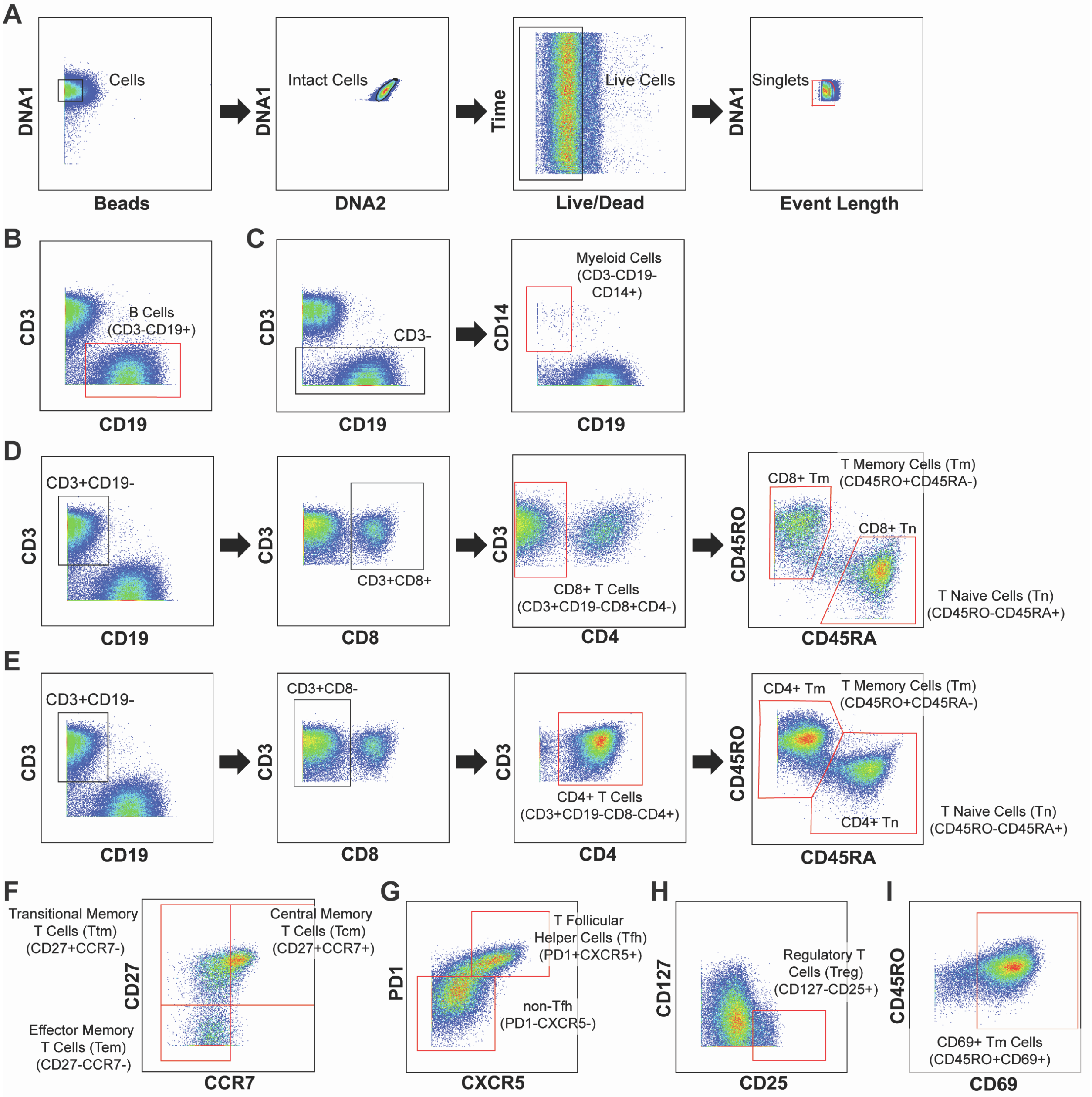

Figure S9. Gating strategies to identify cellular subsets.
Gating strategies were established using tonsillar cells, as they harbor all cellular subsets analyzed in this study (Methods). **(A)** Gating strategy to define live, singlet cells. These cell events were then further gated into B cells **(B)**, myeloid cells **(C),** $CD8^+$ Tm and Tn **(D),** and $CD4^+$ Tm and Tn **(E)**. $CD4^+$ Tm cell events were further gated into Ttm, Tcm, and Tem **(F)**, Tfh and non-Tfh **(G)**, Treg **(H)**, and $CD69^+$ Tm **(I)**. Final gates for each cell subset are indicated in red.

SUPPLEMENTAL TABLES

Table S1. VISORs.

Name	Gene	Category of VISOR	References
Interferon-Induced Transmembrane Protein 1	IFITM1	Restriction Factor	9,10
Polyglutamine Binding Protein 1	PQBP1	Sensor	11,12
Retinoic Acid-Inducible Gene I (DDX58)	RIGI	Sensor	11,12
SAM Domain and HD Domain-Containing Protein 1	SAMHD1/pSAMHD1	Restriction Factor	9,10
Polymerase Associated Factor 1	PAF1	Restriction Factor	13
Toll-Like Receptor 9	TLR9	Sensor	11,12
Absent in Melanoma 2	AIM2	Sensor	14
Interferon Gamma-Inducible Protein 16	IFI16	Sensor/Restriction Factor	11,12
Cyclic GMP-AMP Synthase	cGAS	Sensor	11,12
Stimulator of Interferon Genes	pSTING	Sensing adaptor	11,12
Interferon Regulatory Factor 3	pIRF3	Sensing adaptor	11,12
MX Dynamin-Like GTPase 2	MX2	Sensor/Restriction Factor	10,15
Tripartite Motif Containing 28	TRIM28	Restriction Factor	1,10
Bromodomain-Containing Protein 4	BRD4	Restriction Factor	16
Interferon-Induced Protein with Tetratricopeptide Repeats 1	IFIT1	Restriction Factor	10
Interferon-Induced Protein with Tetratricopeptide Repeats 3	IFIT3	Sensing ISG	10
Schlafen Family Member 11	SLFN11	Restriction Factor	10
MX Dynamin-Like GTPase 1	MX1	Sensing ISG	17

Table S2. VISOR-CyTOF antibodies.

Metal	Antibody	Clone	Catalog	Manufacturer
89Y	CD57	HNK-1	359602	BioLegend
Qdot (112Cd)	HLADR	TU36	Q22158	Invitrogen
141Pr	CD25	M-A251	555430	BD Biosciences
142Nd	CD19	HIB19	3142001B	Standard BioTools
143Nd	IFI16*	1G7	sc-8023	Santa Cruz Biotech
144Nd	CCR5	NP-6G4	3144007A	Standard BioTools
145Nd	IFITM1*	5B5E2	60074-1-Ig	Proteintech
146Nd	CD8	RPA-T8	3146001B	Standard BioTools
147Sm	cGAS*	E5V3W	79978S	Cell Signaling Tech
148Nd	IFIT3*	OT1G1	NBP2-71006	Novus
149Sm	AIM2*	3C4G11	Sc-293174	Santa Cruz Biotech
150Nd	HSA (CD24)	M1/69	3150009B	Standard BioTools
150Nd	LAG3	11C3C65	3150030B	Standard BioTools
151Eu	pSTING*	D8K6H	40818	Cell Signaling Tech
152Sm	SLFN11*	E-4	sc-374339	Santa Cruz Biotech
153Eu	MX2*	H-7	sc-271527	Santa Cruz Biotech
154Sm	IFIT1*	OTI3G8	NBP2-71005	Novus
155Gd	pIRF3*	D6O1M	29047	Cell Signaling Tech
156Gd	SAMHD1*	Polyclonal	12586-1-AP	Proteintech
157Gd	TRIM28*	821912	MAB7785	R and D Systems
158Gd	PAF1*	E-7	sc-514491	Santa Cruz Biotech
159Tb	CCR7	G043H7	3159003A	Standard BioTools
160Gd	MX1*	D3W7I	62815	Cell Signaling Tech
161Dy	CD45RO	UCHL1	304239	BioLegend
162Dy	CD69	FN50	3162001B	Standard BioTools
163Dy	TLR9*	26C593.2	NBP2-24729	Novus
164Dy	CXCR5	RF8B2	3164029B	Standard BioTools
165Ho	pSAMHD1*	T592P	MABF934	Sigma-Aldrich
166Er	PQBP1*	G-12	sc-376039	Santa Cruz Biotech
167Er	CD27	O323	3167002B	Standard BioTools
168Er	PD1	EH12.1	562138	BD Biosciences
169Tm	CD45RA	HI100	3169008B	Standard BioTools
170Er	CD3	UCHT1	3170001B	Standard BioTools
171Yb	RIGI*	4G1B6	NBP2-61849	Novus
172Yb	CD38	HIT2	3172007B	Standard BioTools
173Yb	BRD4*	EPR5150(2)	ab182446	Abcam
174Yb	CD4	SK3	3174004B	Standard BioTools
175Lu	CD14	M5E2	301843	BioLegend
176Yb	CD127	A019D5	3176004B	Standard BioTools
209Bi	TIGIT	MBSA43	3209013B	Standard BioTools

***VISOR and intracellular staining**

Table S3. Last Gift Study participant characteristics.

Person ID	Date of Autopsy	Year of Birth	Sex	Race/ Ethnicity	Terminal Disease	Year of HIV Diagnosis
LG29	February 2022	1976	Male	Caucasian/ Non-Hispanic	Metastatic anal cancer, Kaposi sarcoma	1999
LG31	February 2022	1950	Male	Caucasian/ Hispanic	Diffuse B-cell lymphoma	1985
LG34	September 2022	1992	Male	Caucasian/ Non-Hispanic	Disseminated multidrug resistant MAI, ESRD, cardiac arrest, hyperkalemia	2012
LG28	October 2022	1948	Male	Caucasian/ Non-Hispanic	Metastatic small cell lung cancer	2000
LG22	October 2022	1957	Male	Caucasian/ Non-Hispanic	Cholangiocarcinoma	1992
LG30	December 2022	1960	Male	Black/African American/ Non-Hispanic	HCC, multiple comorbidities	1986
LG33	March 2023	1960	Male	Caucasian/ Non-Hispanic	Esophageal and hepatic cancer	1996

Abbreviations: MAI: Mycobacterium avium infection; ESRD: End-stage renal disease; HCC: Hepatocellular carcinoma;

References

1. Kluge, S.F., Sauter, D., and Kirchhoff, F. (2015). SnapShot: Antiviral Restriction Factors. Cell *163*, 774-774 e771. 10.1016/j.cell.2015.10.019.
2. Cavrois, M., Banerjee, T., Mukherjee, G., Raman, N., Hussien, R., Rodriguez, B.A., Vasquez, J., Spitzer, M.H., Lazarus, N.H., Jones, J.J., et al. (2017). Mass Cytometric Analysis of HIV Entry, Replication, and Remodeling in Tissue CD4+ T Cells. Cell Rep. *20*, 984-998. 10.1016/j.celrep.2017.06.087.
3. Descours, B., Cribier, A., Chable-Bessia, C., Ayinde, D., Rice, G., Crow, Y., Yatim, A., Schwartz, O., Laguette, N., and Benkirane, M. (2012). SAMHD1 restricts HIV-1 reverse transcription in quiescent CD4(+) T-cells. Retrovirology *9*, 87. 10.1186/1742-4690-9-87.
4. Liu, Y., Jesus, A.A., Marrero, B., Yang, D., Ramsey, S.E., Sanchez, G.A.M., Tenbrock, K., Wittkowski, H., Jones, O.Y., Kuehn, H.S., et al. (2014). Activated STING in a vascular and pulmonary syndrome. N. Engl. J. Med. *371*, 507-518. 10.1056/NEJMoa1312625.
5. Gram, A.M., Sun, C., Landman, S.L., Oosenbrug, T., Koppejan, H.J., Kwakkenbos, M.J., Hoeben, R.C., Paludan, S.R., and Ressing, M.E. (2017). Human B cells fail to secrete type I interferons upon cytoplasmic DNA exposure. Mol. Immunol. *91*, 225-237. 10.1016/j.molimm.2017.08.025.
6. Atluri, V.S., Pilakka-Kanthikeel, S., Garcia, G., Jayant, R.D., Sagar, V., Samikkannu, T., Yndart, A., and Nair, M. (2016). Effect of Cocaine on HIV Infection and Inflammasome Gene Expression Profile in HIV Infected Macrophages. Sci. Rep. *6*, 27864. 10.1038/srep27864.
7. Nasr, N., Maddocks, S., Turville, S.G., Harman, A.N., Woolger, N., Helbig, K.J., Wilkinson, J., Bye, C.R., Wright, T.K., Rambukwelle, D., et al. (2012). HIV-1 infection of human macrophages directly induces viperin which inhibits viral production. Blood *120*, 778-788. 10.1182/blood-2012-01-407395.
8. Pallett, L.J., Swadling, L., Diniz, M., Maini, A.A., Schwabenland, M., Gasull, A.D., Davies, J., Kucykowicz, S., Skelton, J.K., Thomas, N., et al. (2023). Tissue CD14(+)CD8(+) T cells reprogrammed by myeloid cells and modulated by LPS. Nature *614*, 334-342. 10.1038/s41586-022-05645-6.
9. Soliman, M., Srikrishna, G., and Balagopal, A. (2017). Mechanisms of HIV-1 Control. Curr. HIV/AIDS Rep. *14*, 101-109. 10.1007/s11904-017-0357-9.
10. Sauter, D., and Kirchhoff, F. (2021). Evolutionary conflicts and adverse effects of antiviral factors. Elife *10*. 10.7554/eLife.65243.
11. Yin, X., Langer, S., Zhang, Z., Herbert, K.M., Yoh, S., Konig, R., and Chanda, S.K. (2020). Sensor Sensibility-HIV-1 and the Innate Immune Response. Cells *9*. 10.3390/cells9010254.
12. Sauter, D., and Kirchhoff, F. (2016). HIV replication: a game of hide and sense. Curr. Opin. HIV AIDS *11*, 173-181. 10.1097/COH.0000000000000233.
13. Kenaston, M.W., and Shah, P.S. (2023). The Archer and the Prey: The Duality of PAF1C in Antiviral Immunity. Viruses *15*. 10.3390/v15051032.
14. Ekabe, C.J., Clinton, N.A., Kehbila, J., and Franck, N.C. (2021). The Role of Inflammasome Activation in Early HIV Infection. J Immunol Res *2021*, 1487287. 10.1155/2021/1487287.
15. Bhargava, A., Lahaye, X., and Manel, N. (2018). Let me in: Control of HIV nuclear entry at the nuclear envelope. Cytokine Growth Factor Rev *40*, 59-67. 10.1016/j.cytogfr.2018.02.006.
16. Rice, A.P. (2019). Roles of CDKs in RNA polymerase II transcription of the HIV-1 genome. Transcription *10*, 111-117. 10.1080/21541264.2018.1542254.
17. Verhelst, J., Hulpiau, P., and Saelens, X. (2013). Mx proteins: antiviral gatekeepers that restrain the uninvited. Microbiol Mol Biol Rev *77*, 551-566. 10.1128/MMBR.00024-13.
